# Structure–Function Relationships of Cid1 Poly(U) Polymerase: Catalysis, Substrate Recognition, Engineering, and Applications

**DOI:** 10.3390/biom16101535

**Published:** 2026-10-21

**Authors:** Yang Chen, Pengbo Wang, Chenglong Zhang, Quanyong Liu, Kian Mau Goh, Shoutao Zhang

**Affiliations:** 1 Faculty of Science, Universiti Teknologi Malaysia, Johor Bahru 81310, Malaysia; chenyang@graduate.utm.my (Y.C.); pengbo@graduate.utm.my (P.W.); 2 School of Life Sciences, Henan Institute of Science and Technology, Xinxiang 453003, China; clzhang@hist.edu.cn (C.Z.); 19213710980@163.com (Q.L.)

**Keywords:** Cid1, poly(U) polymerase, noncanonical poly(A) polymerases, terminal nucleotidyltransferase, uridylyltransferase

## Abstract

RNA 3′-end modification is an important regulatory mechanism that influences RNA processing, stability, localization, function, and degradation. Beyond its biological roles, RNA 3′-end modification has become a valuable tool in biotechnology, enabling applications in RNA labelling, sequencing, engineering and oligonucleotide synthesis. Terminal nucleotidyltransferases are key enzymes responsible for template-independent nucleotide addition to RNA molecules. Among these enzymes, *Schizosaccharomyces pombe* Cid1, a poly(U) polymerase, has emerged as an important model for understanding RNA-specific terminal nucleotidyltransferase activity. Structural and biochemical studies have revealed how nucleotide recognition, metal-ion-dependent catalysis, RNA positioning, and conformational dynamics regulate Cid1-mediated uridylation. Recent advances in protein engineering, molecular simulation, machine learning, and generative protein design have further expanded Cid1 functionality, generating variants with improved activity, altered substrate specificity, and potential applications in enzymatic RNA synthesis. This review summarizes the biological functions, structural characteristics, catalytic mechanisms, protein engineering strategies, and emerging biotechnological applications of Cid1, as well as the remaining challenges, highlighting its potential as a programmable platform for RNA 3′-end functionalization and enzymatic RNA oligonucleotide synthesis.

## 1. Introduction

The terminal nucleotidyltransferase (TENT) family comprises template-independent polymerases that catalyse the addition of non-templated nucleotides to the 3′ ends of nucleic acids. Members of this family, including non-canonical poly(A) polymerases (ncPAPs), terminal uridylyl transferases (TUTs), and poly(U) polymerases (PUPs), exhibit distinct substrate preferences and biological functions (Table 1). The RNA 3′ end serves as an important regulatory element that controls RNA stability, translation efficiency, cellular localization, and decay pathways [1,2,3]. In contrast to template-dependent transcription and canonical polyadenylation, terminal nucleotidyltransferases introduce sequence-independent nucleotide additions that modulate RNA fate [4]. The discovery and characterization of poly(U) polymerases have therefore provided fundamental insights into the role of RNA 3′-end modification in post-transcriptional regulation [5,6,7,8].

Caffeine-induced death suppressor 1 (Cid1) from the fission yeast *Schizosaccharomyces pombe* is a member of the TENT family. The protein was named following its identification in a genetic screen for mutants affecting cytokinesis and caffeine sensitivity [3,6,7,58,59]. Initially, Cid1 was considered a poly(A) polymerase (PAP)-like enzyme because it contains a PAP-associated domain, also known as the Cid1-family poly(A) polymerase domain (PF03828), together with a nucleotidyltransferase domain (PF01909), and early in vitro studies detected poly(A) polymerase activity [38,59]. The coexistence of these domains places Cid1 within the broader family of RNA-specific ribonucleotidyl transferases and supports its evolutionary relationship with both canonical and non-canonical terminal nucleotidyltransferases [8,60].

However, subsequent biochemical and cellular studies established that Cid1 was not a physiological poly(A) polymerase. Instead, this enzyme preferentially utilizes UTP and is now recognised as one of the well-characterised poly(U) polymerases (EC 2.7.7.52) involved in RNA 3′-end uridylation [5,7]. This discovery positioned Cid1 as both a model enzyme for understanding the biological functions of RNA uridylation and a representative system for investigating the catalytic mechanisms of template-independent RNA terminal nucleotidyl transfer [9,61,62,63]. Structural and biochemical studies have revealed that Cid1 achieves UTP selectivity through a combination of nucleotide recognition, RNA 3′-end positioning, and dynamic conformational changes rather than through a single determinant [9,61,62,63]. These studies include landmark studies that have made significant contributions to our current understanding of Cid1 structure, catalytic mechanism, and biological function.

Cid1 has gained increasing attention beyond its native role in RNA uridylation, with emerging applications in RNA engineering and biotechnology [64,65,66], including nanopore-based RNA sequencing and functional 3′-end RNA engineering. Furthermore, engineered Cid1 variants combined with 3′-O-blocked reversible terminator nucleotides have demonstrated the potential for template-independent RNA oligonucleotide synthesis through iterative extension cycles [65]. Recent advances in computational protein engineering, integrating molecular dynamics simulations, machine learning, and generative protein design, have enabled the rational optimization of Cid1, leading to improvements in catalytic activity, structural stability, and substrate compatibility [66]. Together, these advances highlight the potential of Cid1 as a programmable platform for RNA 3′-end functionalisation and enzymatic RNA synthesis.

In recent years, several reviews have discussed enzymes within the TENT family [7,8,47,52,67,68]; however, comprehensive reviews focusing specifically on Cid1 remain limited. The most recent review dedicated to Cid1 was published in 2008 [7], and subsequent advances in structural studies, protein engineering, and biotechnological applications have generated significant new insights that warrant an updated review article. This report summarizes the structural basis, catalytic mechanism, and substrate selectivity of Cid1, with particular emphasis on the key residues and dynamic structural elements that govern RNA uridylation. We further discuss emerging applications of Cid1, including template-independent enzymatic RNA synthesis, RNA sequencing, RNA labelling and bioconjugation and functional RNA engineering. Finally, this review highlights current challenges and future perspectives in Cid1 engineering, including improving substrate generality, optimizing reversible terminator chemistry, and expanding compatibility with chemically modified nucleotides.

## 2. Cid1 Background

### 2.1. Historical Discovery of Biological Roles

**Figure 1:**
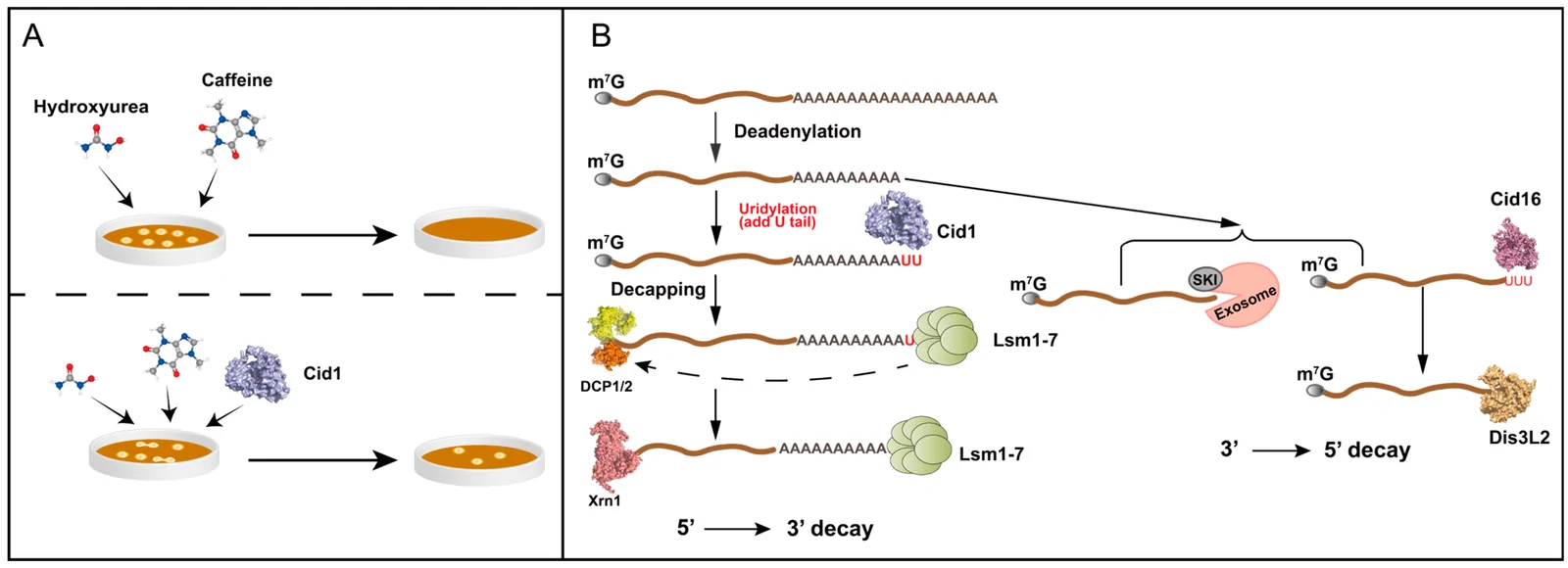
Functional roles of Cid1. (**A**) Functional roles of Cid1 in S-M checkpoint. (**B**) Model of bulk mRNA decay in fission yeast. Grochowski [3] proposed that uridylation plays a dual role in bulk mRNA turnover. Cid1 uridylates shortened poly(A) tails, promoting Lsm1-7 binding and directing transcripts toward 5′ to 3′ decay. This uridylation also protects the 3′ end from excessive deadenylation and thereby suppresses 3′ to 5′ decay of partially deadenylated mRNAs. Once transcripts become fully deadenylated, they can either be degraded by the exosome or oligouridylated by Cid16, which targets them for Dis3L2-dependent U-tail-mediated decay. Solid arrows indicate sequential steps and pathways of mRNA decay, whereas the dashed arrow indicates Lsm1–7-mediated stimulation of decapping. Red U residues indicate the uridylated RNA tails.

Cid1 was first identified in *S. pombe* in 2000 in a genetic screen designed to identify factors involved in cell survival following combined treatment with hydroxyurea and caffeine (Figure 1A). Subsequent genetic studies implicated Cid1 in the S-phase checkpoint response to impaired DNA replication, while sequence analysis suggested that Cid1 belongs to the nucleotidyltransferase family [59]. In 2002, Cid1 was shown to use RNA substrates and exhibit cytoplasmic poly(A) polymerase activity, leading to its initial classification as a poly(A) polymerase [38].

This classification was revised in 2007 when biochemical studies demonstrated that Cid1 preferentially utilizes UTP and functions primarily as an RNA terminal uridylyltransferase, also known as a poly(U) polymerase [5,6]. In 2009, Cid1-dependent uridylation was detected at the 3′ ends of polyadenylated mRNAs, including act1, hcn1, and urg1. Uridylation preceded and appeared to stimulate decapping, and was proposed to promote Lsm1-7-associated recruitment of the decapping machinery [58]. In 2013, the discovery of the uridine-preferring 3′-5′ exoribonuclease Dis3L2 in *S. pombe* raised the possibility that some uridylated RNAs could also enter an alternative 3′-5′ surveillance pathway [69]. However, these studies did not establish Dis3L2 as an obligatory downstream nuclease for Cid1-modified transcripts. Transcriptome profiling in 2019 further suggested that the relatively modest consequences of Cid1 deletion could reflect compensation by other terminal uridylyltransferases [70].

Transcriptome-wide analysis in 2023 demonstrated that mRNA uridylation is widespread in fission yeast and identified Cid1 as the primary uridyltransferase responsible for this modification, with Cid16 contributing additional activity [11]. Subsequent 3′-end profiling published in 2024 provided further mechanistic insight, showing that Cid1 preferentially adds one or two uridine residues to shortened poly(A) tails. This uridylation promotes Lsm1-7-associated decapping and subsequent 5′-to-3′ mRNA decay, while limiting excessive deadenylation and the subsequent entry of transcripts into exosome-mediated 3′-to-5′ degradation [3]. The two routes differ in their initiating RNA end and enzymatic machinery: Lsm1–7 promotes decapping, after which Xrn1 degrades the RNA from its 5′ end, whereas the Superkiller (SKI) complex-assisted RNA exosome degrades extensively deadenylated transcripts from their exposed 3′ ends [3,58]. When Cid1-mediated uridylation is impaired, the contribution of 3′-to-5′ decay increases, although the relative decay rates of the two routes have not been directly established [3]. Completely deadenylated RNAs may instead undergo Cid16-dependent oligouridylation and subsequent Dis3L2-mediated decay, although this branch remains a proposed model rather than a fully resolved pathway (Figure 1B) [3]. Collectively, Cid1 is now recognised as the principal poly(A)-tail uridyltransferase that contributes to mRNA decay directionality in *S. pombe*.

### 2.2. Cid1 Family and Related Terminal Nucleotidyltransferase (TENT) Family

Historically, six proteins in *S. pombe* were grouped within the Cid1 family based on sequence similarity and their shared Pol β-like nucleotidyltransferase architecture [8,38]. Subsequent studies, however, revealed that these proteins have diverged considerably in their substrate specificities, cellular localizations, and biological functions. Cid1 and Cid16 primarily exhibit uridylyltransferase activity [5,6,11,12], whereas Cid13 and Cid14 mainly function in adenylation [42,46]. Cid12 has been incorporated into the RNA interference machinery [40,71]. Cid11 is annotated as a poly(A) RNA polymerase, although it remains insufficiently characterized [72]. Among these proteins, Cid1 represents a structurally compact and well-characterized poly(U) polymerase within the TENT superfamily, with a pronounced preference for UTP [6,61,62].

**Figure 2:**
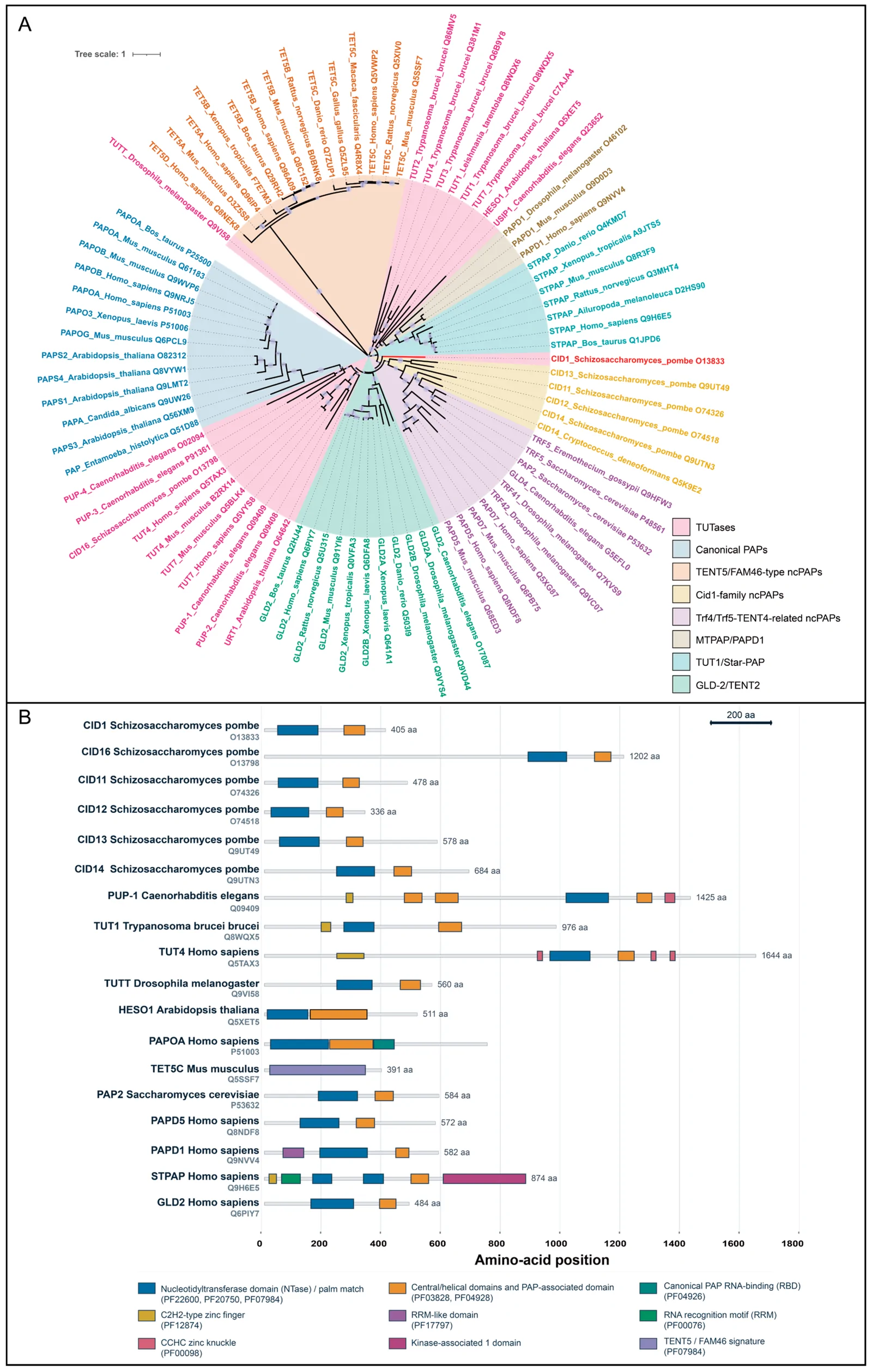
Phylogenetic context and domain architectures of the *S. pombe* Cid1 family and related RNA terminal nucleotidyltransferases. (**A**) Comparative protein-sequence phylogeny. *S. pombe* Cid1 is highlighted in red. Eight annotation groups are colour-coded: TUTases; canonical PAPs; TENT5/FAM46-type non-canonical PAPs; Cid11–Cid14 homologues; Trf4/Trf5–TENT4-related non-canonical PAPs; MTPAP/PAPD1; TUT1/Star-PAP; and GLD-2/TENT2. These colours identify named protein groups and do not imply that all groups are monophyletic or have identical nucleotide preferences. Protein sequences were aligned using MUSCLE [73] as implemented in MEGA12 (version 12.0.15; Institute for Genomics and Evolutionary Medicine, Temple University, Philadelphia, PA, USA), and the phylogenetic tree was inferred using the maximum-likelihood method in MEGA12 [74]. The resulting phylogenetic tree was visualized and annotated using Interactive Tree of Life (iTOL, version 7.6; European Molecular Biology Laboratory, Heidelberg, Germany) [75]. (**B**) Comparative domain-region maps of 18 representatives, including all six *S. pombe* Cid proteins. Grey bars indicate full-length sequences; positions and lengths are drawn on a common amino-acid scale. Coloured rectangles indicate distinct functional domains: blue, NTase/palm domain; orange, PAP-associated domain; teal, the canonical PAP RNA-binding domain (RBD); gold, C2H2-type zinc fingers; purple, an RRM-like (RL) domain; green, an RNA recognition motif (RRM); pink, CCHC zinc knuckles; and magenta, a kinase-associated 1 (KA1) domain.

Although TENT-family proteins share the common ability to catalyse template-independent nucleotide addition, these enzymes display substantial diversity at both the sequence and structural levels. Individual members differ considerably in protein length, sequence identity, domain organization, and conserved motifs, corresponding to their distinct substrate preferences and biological functions (Table 1, Figure 2) [47,52,60]. A phylogenetic tree was constructed using representative protein sequences and Cid1 (Figure 2A). The sequences were assigned into eight groups based on their annotations, with different colours used to facilitate comparison. This distinction is important because PAP, TUTase, and PUP are primarily functional designations rather than mutually exclusive phylogenetic classifications. In particular, PUPs are TUTase capable of catalysing polyuridylation, while closely related TENTs may exhibit different nucleotide preferences [5,72]. We subsequently run Interpro to identified domains and key features for all these sequences, and few of these are shown in Figure 2B. Cid1 exhibit only nucleotidyltransferase (NTase) domain (PF01909) and a PAP-associated region (PF03828), which is relatively simpler than some proteins. For example, STPAP, selected from the TUT1/Star-PAP group, contains not only these two domains but also additional accessory domains. These differences in domain architecture help explain the diversity in substrate preferences and biological functions among TENT-family proteins.

### 2.3. Nucleotide Substrates of Cid1

Cid1 adds nucleotides to the 3′ end of an RNA molecule without requiring a template to specify the sequence. The enzyme uses nucleoside triphosphates as the source of the added nucleotides. Although Cid1 primarily functions as a poly(U) polymerase in vivo, biochemical and structural studies have demonstrated broader, but highly unequal, nucleotide-substrate tolerance [6,9,64,76]. Its active-site cleft can accommodate all four canonical ribonucleoside triphosphates (rNTPs)—ATP, GTP, CTP, and UTP—which provide the building blocks for adding adenine (A), guanine (G), cytosine (C), and uridine (U), respectively (Table 2) [38,65]. Although Cid1 can accommodate all four rNTPs, it strongly favours UTP over the other substrates. Kinetic analyses showed that Cid1 achieves half-maximal activity at substantially lower UTP concentrations and incorporates UTP more rapidly and efficiently than ATP under the assay conditions [9]. GaMD/MM-PBSA simulations further supported this preference, predicting Cid1 binding affinity in the order UTP > ATP > CTP > GTP [76]. Although this ranking differs somewhat from the biochemical results, which showed weak GTP incorporation but no detectable CTP incorporation under their assay conditions [9], both approaches indicate that UTP and ATP are more favourable substrates than CTP and GTP [9,76].

The broad substrate tolerance of Cid1 also raises the question of how chemical modifications to nucleotides affect substrate recognition and catalysis. Beyond the four canonical rNTPs, studies have therefore examined Cid1 activity toward chemically modified nucleotide substrates, revealing how changes to the nucleobase or ribose moiety influence substrate binding and catalytic efficiency. Wild-type Cid1 efficiently polymerizes inosine triphosphate (ITP) to generate poly(I) tails and accepts the base-modified uridine analogues 5-methyl-UTP and 4-thio-UTP (Table 2), whereas pseudouridine triphosphate and N^1^-methylpseudouridine triphosphate were not detectably polymerized under the tested conditions [64]. Cid1 can also use N^6^-(6-aminohexyl)-ATP–biotin, producing RNA carrying multiple or long biotinylated tails. In contrast, 2′-azido-2′-deoxy-NTPs are incorporated only with moderate efficiency and generally yield heterogeneous products, while significant incorporation of the base-modified analogue 8-azido-ATP or 3′-azido-modified dideoxynucleotides was not observed [77,80].

Cid1 also accepts several C5-functionalized UTP analogues carrying fluorescent or bioorthogonal groups. The heterocycle-modified fluorescent analogues 5-selenophene-UTP (SeUTP) and 5-benzofuran-UTP (BFUTP) can serve as substrates for Cid1. Under the reported conditions, BFUTP supports multiple nucleotide additions, whereas SeUTP results in only one or a few additions (Table 2). Both substrates have been used as environment-sensitive fluorescent nucleotide probes to facilitate estimation of the binding affinities of the enzyme for RNA and nucleotides using fluorescence spectroscopy [78]. Cid1 also incorporates the C5-azide-modified substrates 5-azidomethyl-UTP (AMUTP), 5-azidopropyl-UTP (APUTP), and an azide-modified UTP bearing a tetraethylene-glycol spacer (ATUTP) [79]. AMUTP supports repeated addition and has been used to generate azide-tailed CRISPR guide RNAs, whereas the longer C5 substituents generally favour fewer incorporation events [79]. Subsequent control of the metal-ion balance or addition of borate enabled single-nucleotide incorporation of SeUTP and BFUTP in moderate yields and of the clickable AMUTP, APUTP and ATUTP in higher yields [85]. More recently, a glycine-modified uridine triphosphate, designated GUTP, was also shown to serve as a Cid1 substrate, allowing a Gly-tag to be installed at the RNA 3′ terminus for subsequent chemoselective conjugation [84]. These findings establish the solvent-exposed C5 position of uracil as a permissive site for introducing fluorescent, azide, alkyne, and amino-acid-derived functionalities without abolishing Cid1-mediated transfer.

The sugar tolerance of Cid1 extends beyond conventional ribonucleotides. Commercial Cid1 incorporated unblocked locked-nucleic-acid thymidine triphosphate (LNA-TTP) and several 3′-O-protected DNA and LNA thymidine triphosphates into RNA primers with approximately 80–95% conversion to N + 1 products [81]. Unblocked LNA-TTP itself behaved as a chain terminator, preventing further extension even though its 3′-hydroxyl group was available. However, the 3′-O-azidomethyl group of the LNA analogue was lost during or after incorporation [81]. LNA-based sugar functionalization was further extended to terminal labeling: commercial NEB PUP incorporated 3′-triGalNAc-LNA-TTP into a natural RNA primer, yielding an N + 1 conjugate bearing a bulky triantennary N-acetylgalactosamine (triGalNAc) ligand at the sugar 3′ position [83]. Under the tested conditions, a fully 2′-O-methylated RNA primer could not be extended even with unmodified dTTP [83]. For controlled RNA synthesis, commercial Cid1 also accepted selected 2′,3′-O-protected ribonucleotides: acetal-protected UTPs predominantly yielded N + 1 products under optimized conditions, with isopropylidene-protected UTP and CTP supporting efficient single additions, whereas protected purines were less effective or produced mixed products [82]. Only partial acidic deprotection of the isopropylidene-protected RNA product was achieved, leaving a complete iterative extension–deblocking cycle undemonstrated [82]. These findings show that efficient single-nucleotide incorporation does not necessarily translate into iterative synthesis, which additionally requires stable termination and compatible deblocking (Table 2) [81,82].

Protein engineering further broadens Cid1 substrate utilization and enables iterative synthesis when paired with suitable reversible-terminator and deblocking chemistry. Structural and mutational studies identified H336 as a major determinant of uracil recognition, and substitution of this residue with arginine, either alone or in combination with N171A and T172S, reduced the strong UTP preference of wild-type Cid1 and enhanced acceptance of alternative nucleotide substrates [9,61,65]. The resulting variants supported incorporation of all four 3′-O-allyl reversible-terminator NTPs and were compatible with selected 2′-fluoro, 2′-O-methyl-, deoxy-, and α-phosphorothioate-modified nucleotides (Table 2) [65]. Using 3′-O-allyl RT-NTPs, an engineered Cid1 platform demonstrated ten full extension–deblocking cycles in liquid-phase RNA synthesis, with an average coupling efficiency of approximately 95% [65]. They also incorporated 3′-O-propargyl-NTPs to generate discrete alkyne-functionalized N + 1 products, whereas unblocked N^6^-propargyl-ATP and 2-ethynyl-ATP underwent uncontrolled polymerization to form modified homopolymer extensions longer than 100 nucleotides [65]. Selected 3′-O-azidomethyl reversible terminators were also incorporated and deblocked, although compatibility was not demonstrated uniformly for all four nucleobases [65]. Collectively, these findings establish engineered Cid1 variants as catalysts for controlled RNA synthesis when nucleotide recognition, reversible termination, and chemical deblocking are jointly compatible [65].

### 2.4. RNA Substrates of Cid1

Unlike template-dependent polymerases, Cid1 does not read sequence information from the RNA substrate. Instead, the RNA molecule serves as a primer that provides an accessible 3′-hydroxyl group for nucleotide addition [6,60]. In vivo, Cid1 preferentially uridylates mRNAs with shortened poly(A) tails, generally adding one or a few uridines that influence their subsequent entry into RNA-decay pathways [3,11,58]. In vitro, however, Cid1 exhibits a broader RNA-substrate tolerance. Reported primers include short homopolymeric oligonucleotides, the minimal ApU pseudo-product, total yeast tRNA, cellular RNA preparations, pre-let-7a, mature miRNAs, polyadenylated mRNAs, rRNAs and diverse non-polyadenylated RNAs [10,64,70]. Nevertheless, extension efficiency remains substrate dependent; for example, tRNAs and 5S rRNA are inefficiently poly(I)-tailed within complex total-RNA samples [64]. Although Cid1 does not strictly require a specific RNA sequence or defined secondary structure, RNA length, terminal nucleotide composition, 3′-end accessibility, and the identity of the incoming nucleotide influence substrate utilization and the mode of polymerization [6,10,64,70].

In controlled oligoribonucleotide synthesis, the RNA primer is commonly termed an initiator. Initiators of at least approximately ten nucleotides can be homopolymeric or rationally designed [65]. Engineered Cid1 variants preferentially extend RNA initiators, although DNA-containing initiators can also be accepted when a terminal ribonucleotide providing a 2′-hydroxyl group is positioned at the 3′ end [65]. Overall, Cid1 is best described as a UTP-preferential RNA 3′-end nucleotidyltransferase whose activity is jointly governed by the chemical structure of the nucleotide donor and the terminal context of the RNA primer.

## 3. Structural Basis and Catalytic Mechanisms of Cid1

### 3.1. CAT Domain

**Figure 3:**
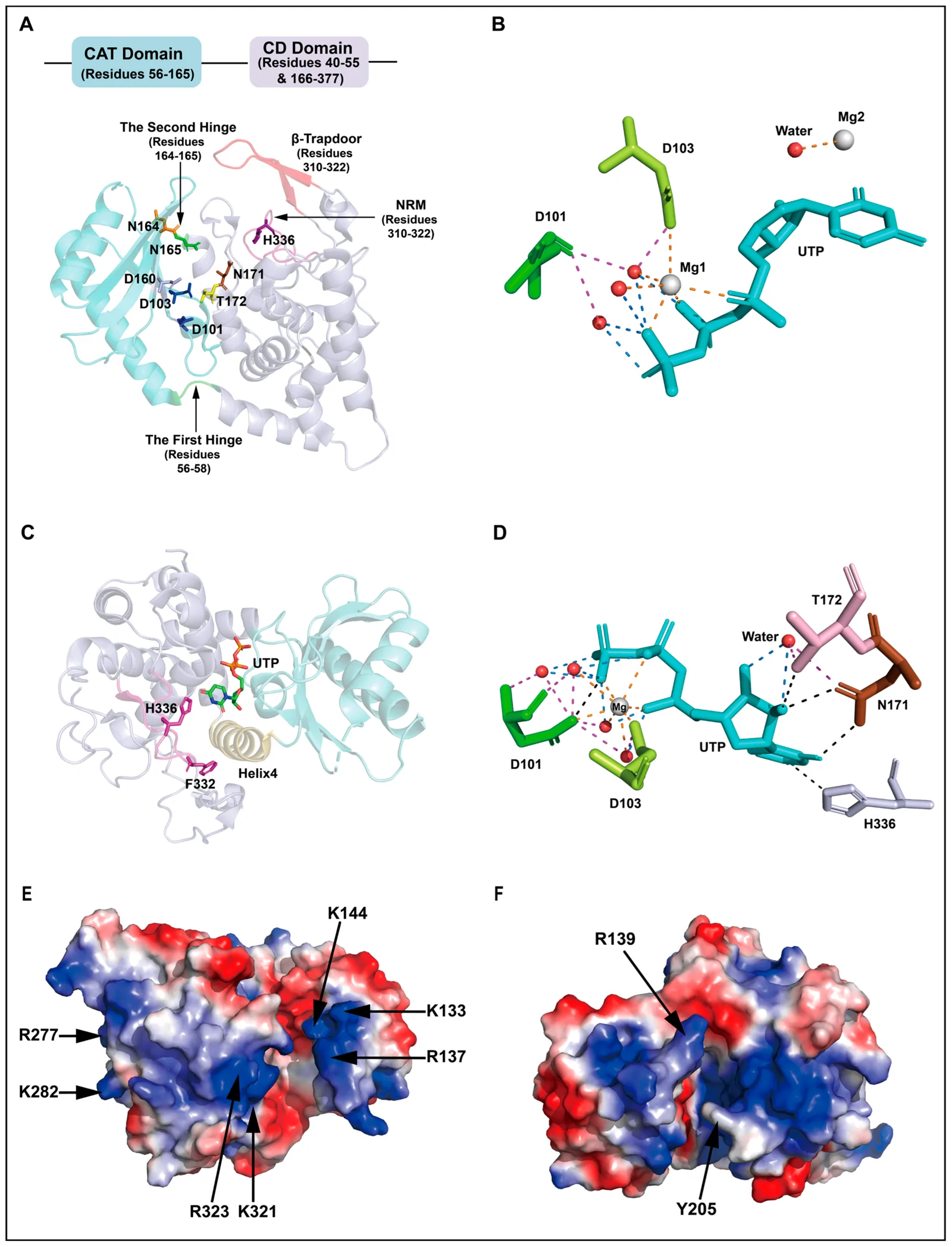
Structural basis of Cid1-mediated UTP recognition and RNA 3′-end uridylation. (**A**) Overall Cid1 structure (PDB 4UD5), showing the CD (pale lavender), CAT (light cyan), β-trapdoor (red), NRM (magenta), D101 (Blue), D103 (Blue), D160 (light purple), N164 (orange), N165 (green), N171 (brown), T172 (yellow) and H336 (magenta). (**B**) Mg^2+^-associated contact network (PDB 4EP7), showing UTP (cyan), D101 (green), D103 (lime green), two Mg^2+^ ions (gray spheres), and selected water molecules (red spheres). (**C**) Alternative view of Cid1 (PDB 4EP7), showing helix 4 (yellow) and H336 and F332 (magenta sticks). (**D**) UTP-recognition network (PDB 4FH5), showing direct and water-mediated contacts involving D101 (green), D103 (lime green), N171 (brown), T172 (pink), and H336 (pale lavender). UTP, Mg^2+^, and water molecules are colored as in (**B**). (**E**) Electrostatic surface of Cid1 (PDB 4E80; blue, positive; red, negative), showing the putative RNA-binding surface, with K133, R137, K144, R277, K282, K321, and R323 indicated. (**F**) Electrostatic surface of the Cid1 active-site cleft (PDB 4EP7, colored as in E), with R139 and Y205 indicated. In panel (**B**,**D**), orange, blue, and magenta dashed lines indicate Mg^2+^-associated contacts, UTP–water contacts, and residue–water contacts, respectively; black dashed lines in D indicate direct UTP–residue contacts. All structures were rendered using Open-Source PyMOL (The PyMOL Molecular Graphics System, version 3.2.0a; Schrödinger, LLC, New York, NY, USA).

Cid1 consists of 405 residues with an approximate molecular mass of 46 kDa [62]. Its catalytic (CAT) domain (residues 56–165) comprises a five-strand β-sheet flanked by two α-helices and forms one side of the nucleotide-binding cleft (Figure 3A) [9]. Together with the central domain, the CAT domain provides the structural framework required for nucleotide binding, RNA-primer positioning, and metal-ion-dependent nucleotidyl transfer [6,9,10,61,62,63].

The CAT domain contains the highly conserved aspartate residues D101, D103, and D160, which form the catalytic triad characteristic of Pol β-like nucleotidyltransferases [6,10,61,62]. D101 and D103 directly participate in divalent-metal coordination (Figure 3D), whereas D160 is also indispensable for catalytic activity. Consistent with their essential roles, substitution of D101 and D103 with alanine (D101A/D103A) or D160 with alanine (D160A) abolishes nucleotide transfer [6,10]. However, the D160A mutant retains RNA-binding affinities for the tested A15 and U15 substrates comparable to those of wild-type Cid1, indicating that disruption of the catalytic residue does not substantially impair RNA binding [10].

Available Cid1 structures support a metal-dependent catalytic mechanism but differ in the number and position of resolved divalent cations. In the UTP-bound structure PDB 4EP7, two Mg^2+^ ions were observed adjacent to the catalytic site (Figure 3B) [61]. One Mg^2+^ ion is coordinated by D103 and phosphate oxygen atoms of the bound nucleotide, whereas the second Mg^2+^ is positioned near the uracil base and the expected location of the RNA 3′ end [61]. In contrast, the Cid1–MgUTP structure PDB 4FH5 contains only a single divalent cation coordinated by D101, D103, the UTP triphosphate group, and water molecules (Figure 3D) [9]. Therefore, although the second Mg^2+^ observed in 4EP7 may contribute to RNA substrate positioning, its precise catalytic role and physiological relevance remain unresolved.

The ApU pseudo-product-bound structure (PDB 4NKU) provides a clue to how Cid1 distinguishes between RNA and DNA substrates. In this structure, the 2′-hydroxyl group of the penultimate adenosine ribose forms a direct hydrogen bond with D103 [10]. Because this hydroxyl group is present in RNA but absent from DNA, this interaction provides a plausible structural explanation for Cid1′s preference for RNA. However, this observation comes from a D160A-containing pseudo-product complex rather than a fully catalytic ternary complex. It is therefore best viewed as structural evidence supporting a model of RNA recognition rather than as direct evidence of the catalytic mechanism.

Mutagenesis studies further indicate that several residues surrounding the catalytic pocket contribute to Cid1 activity, although not necessarily through direct RNA contacts. R139 lies near the entrance of the catalytic pocket (Figure 3F) and occupies a position analogous to R121 in *Trypanosoma brucei* TUT4, where the corresponding residue contributes to primer stabilization [10]. Although activity assay indicated that R139A reduced polymerase activity, subsequent structural and binding analyses suggested that R139 does not directly contact the ApU pseudo-product and that R139A binds A15 and U15 substrates similarly to wild-type Cid1 [10,61]. These findings suggest that R139 influences catalysis indirectly, rather than serving as a major RNA-recognition residue. Y205 provides another example of a residue whose contribution appears to extend beyond direct substrate binding. Located opposite R139 in the CD domain near the entrance to the catalytic pocket (Figure 3F), Y205 is important for efficient catalysis, with several substitutions reducing activity and Y205D almost completely abolishing it (Table 3) [61]. However, no direct interaction between Y205 and the RNA substrate has been established. Its effect is therefore more likely to arise from changes in the local active-site architecture or from conformational coupling that affects productive catalysis.

K144 appears to have a more substrate-dependent role. The K144A mutation substantially reduces the productive use of an A15 primer while having a much smaller effect on U15 [10]. Despite this difference, K144A retains near-wild-type poly(U) polymerase activity under the tested conditions, whereas ATP-dependent extension is strongly impaired and becomes predominantly distributive. The weaker effect reported in an earlier study using a longer RNA substrate further suggests that the contribution of K144 may depend on primer length, sequence, and assay conditions [10,61,62].

Mutational studies aimed at improving Cid1 performance provide a complementary perspective on the CAT domain. In the PUPdel engineering study, residue numbering differs from full-length Cid1 by 12 positions because residues 1–12 were deleted [66]. Thus, C63I, D97P, I128V, and K132R in PUPdel correspond to C75I, D109P, I140V, and K144R in full-length Cid1, respectively. These substitutions highlight that improvements in enzyme performance do not necessarily arise from directly modifying the catalytic residues. C75I, D109P were associated mainly with improved local structural stability or thermostability, whereas K144R was proposed to strengthen a local electrostatic network [66]. I140V produced a modest improvement in extension with unmodified NTPs compared to PUPdel. Collectively, these findings illustrate that the CAT domain functions as an integrated structural and catalytic unit, in which residues outside the immediate catalytic center can influence activity by altering local stability, electrostatic interactions, or the conformational environment of the active site.

### 3.2. CD Domain

The central domain (CD domain) is composed of two discontinuous polypeptide segments (residues 40–55 and 166–377), which together form a structure containing six α-helices and two short β-strands connected by loops [9]. Together with the CAT domain, the CD domain forms the catalytic cleft that accommodates both the incoming nucleotide and the 3′ terminus of the RNA substrate (Figure 3A). Notably, the C-terminal portion (residues 166–377) of the CD domain is particularly important for nucleotide recognition, substrate positioning, and catalytic conformational changes discussed below.

Structural studies of Cid1 bound to UTP (PDB 4E80 and 4FH5) highlight N171 as an important nucleotide-recognition residue [9,62]. In the MgUTP-bound structure (PDB 4FH5), the side-chain carbonyl group interacts with the ribose 2′-hydroxyl group, whereas its amino group contacts the O2 carbonyl of the pyrimidine base (Figure 3D) [9]. The side-chain hydroxyl of T172 is also positioned to hydrogen-bond with the ribose 2′-hydroxyl group and contacts the 3′-hydroxyl group through a bridging water molecule [9]. These contacts coexist in the observed UTP-bound structure; however, functional cooperativity between N171 and T172 has not been established [9]. The dual ribose–base interaction formed by N171 with UTP is proposed to contribute to ribose recognition and discrimination against dUTP; structural comparisons indicate that N171 can form the same interaction with CTP but not purine nucleotides, suggesting that its primary role is in pyrimidine recognition rather than uracil-specific recognition [62].

Residues 327–339 constitute the nucleotide-recognition motif (NRM), which contains several of the principal determinants of nucleotide selectivity [9]. H336 occupies a central position within this motif. In the Cid1-MgUTP structure (4FH5), the imidazole side chain of H336 forms a direct hydrogen bond with the O4 carbonyl group of the uracil base, contributing to UTP recognition and positioning within the nucleotide-binding pocket [9]. H336 can adopt alternative conformations and participates in an extended recognition network involving neighbouring residues and water-mediated interactions [62,63]. The functional importance of H336 is supported by mutational analyses. Replacement with asparagine (H336N) reduces UTP preference and increases the relative incorporation of ATP [9,63]. In contrast, substitution with alanine (H336A) shifts the relative substrate preference toward PAP-like activity in some assays, although other studies reported reduced catalytic activity with both ATP and UTP [61,62]. Likewise, H336L decreases activity toward both nucleotides while retaining a greater preference for UTP than ATP [61]. The importance of this position is further supported by studies of other TENT enzymes. Human GLD2/TENT2 normally favours ATP, but introduction of a histidine at the structurally corresponding position enhances UTP incorporation and decreases ATP preference [86]. Together, these findings demonstrate that a single residue within the nucleotide-recognition motif can profoundly influence nucleotide selectivity, suggesting that remodeling a limited number of recognition residues is sufficient to shift the balance between PAP- and PUP-like activities during the evolution and engineering of TENT enzymes.

Other residues within the NRM contribute to the H336-centred recognition network, including D330 and E333. D330A mutant reduces poly(U) polymerase activity, whereas E333A mutation causes a much stronger reduction in PUP activity [62]. These findings suggest that D330 and E333 contribute to nucleotide recognition as part of a coordinated network rather than acting as independent determinants of base specificity. F332, another residue within the NRM, appears to play a different but complementary role. This residue is proposed to connect the NRM loop to helix 4, with NTP binding inducing a rotation of helix 4 that positions F332 within a hydrophobic pocket and helps stabilize the nucleotide-recognition conformation (Figure 3C) [10,61]. Mutational studies support the importance of this hydrophobic environment: substitutions that retain some hydrophobic character, such as F332A and F332L, preserve substantial catalytic activity, whereas replacement with the charged residue F332D severely impairs catalysis [61]. In addition, the F332A variant exhibits reduced RNA-binding capacity, suggesting that F332 contributes to maintaining the structural environment required for efficient RNA binding and catalysis [10]. Thus, the effects of F332 mutations are unlikely to result solely from altered positioning of the NRM loop.

Engineering residues within the CD-domain region of the nucleotide-binding cleft has substantially broadened the substrate scope of Cid1. Wiegand et al. showed that the H336R and H336R/N171A/T172S variants enabled template-independent incorporation of all four 3′-O-allyl reversible-terminator NTPs (Table 3) [65]. However, because N171A and T172S were introduced together with H336R, their individual contributions to the improved incorporation of modified nucleotides could not be clearly distinguished. The PUPdel study systematically screened mutations within the active site and identified several variants that improved the incorporation of selected 3′-O-allyl-NTPs and some were predicted to enhance local structural stability (Table 3) [66]. These studies demonstrate that the nucleotide-binding cleft is not only important for substrate recognition but also provides a useful target for engineering Cid1 substrate scope and performance.

### 3.3. β-Trapdoor

The CD domain contains a flexible β-trapdoor spanning residues 310–322. This element forms a short, two-stranded antiparallel β-sheet located near the interface of CAT and CD domains, where it partially covers the catalytic cleft [10,61,62,63]. This β-trapdoor undergoes conformational changes during catalysis and contributes to substrate containment, product release, and resetting of the active site between successive catalytic cycles [10,61,62,63]. Furthermore, β-trapdoor may stabilize the nucleotide-bound state and prevent premature collapse of the cleft following UTP–Mg^2+^ binding [63]. Deletion of the whole stretch of residues does not substantially alter the binding affinity of Cid1 for the tested RNA substrates and does not abolish overall UTP-dependent poly(U) polymerase activity [10]. However, when ATP is supplied as the incoming nucleotide, the Δ310–322 mutant produces few or no long extension products and displays predominantly distributive PAP activity [10]. These findings indicate that the β-trapdoor is not essential for RNA binding or basal UTP-dependent catalysis. Instead, it appears to modulate nucleotide-dependent conformational changes that influence the efficiency and mode of repeated nucleotide addition.

The dynamic properties of the β-trapdoor have also made this region an attractive target for protein engineering. The engineered PUPdel2 variant contains 16 substitutions distributed throughout the protein, including A297R, S303R, and Q306S in truncated- PUPdel numbering [66], which corresponded to A309R, S315R, and Q318S in the full-length sequence. Among these substitutions, S315R and Q318S are located within the β-trapdoor region, whereas A309R is located immediately adjacent to its N-terminal edge. The introduction of arginine residues at A309 and S315 increases the positive electrostatic character of the β-trapdoor and adjacent substrate-entry region. Molecular simulations further suggested altered flexibility in this region, leading to the proposal that the engineered β-trapdoor acts as a dynamic “attractive gateway” that facilitates RNA-primer recruitment and access to the catalytic cleft [66].

### 3.4. Interdomain Hinges

The CAT and CD domains are connected by two loop hinge regions centred around residues 56–58 and 164–165 [9,61]. These flexible regions allow the two domains to move relative to each other and undergo conformational changes during nucleotide binding, catalysis, RNA-product repositioning, and nucleotide exchange [62,63]. Their importance is also supported by mutational and protein-engineering studies [63,66]. The first hinge (residues 56–58) has been targeted for engineering Cid1 activity. In the engineered PUPdel scaffold, I45 corresponds to I57 in the full-length Cid1 sequence. The I57P substitution increased catalytic efficiency toward 3′-O-allyl-CTP by approximately fourfold and improved thermostability [66]. Although the structural basis of these effects remains unclear, introducing proline at this position may alter local hinge flexibility and, consequently, the relative movement of the CAT and CD domains.

The second hinge, comprising residues 164–165, appears to have a more direct role in RNA-product repositioning and iterative catalysis. In the ApU pseudo-product-bound structure (PDB 4NKU), N165 is positioned near the RNA 3′ terminus and forms a hydrogen bond with the N1 atom of the penultimate adenosine [10]. The N165A substitution does not substantially alter the binding affinity of Cid1 for the tested A15 or U15 substrates but restricts extension predominantly to a single nucleotide under both ATP- and UTP-dependent conditions. In contrast, N165D permits the addition of several uridines or a longer adenine tract to a 15-nucleotide RNA primer but almost abolishes extension of the short ApU substrate [10]. These findings indicate that N165 contributes to catalytic cycling in a manner dependent on primer length, terminal sequence, and incoming nucleotide identity. The neighbouring hinge residue N164 also influences repeated nucleotide addition. The N164P mutation produces predominantly monouridylated products, whereas the N164P/N165P double mutation causes a strong reduction in terminal uridylyltransferase activity [63]. Together, these findings indicate that the interdomain hinges function as dynamic regulatory elements rather than passive linkers, maintaining the conformational flexibility required for substrate accommodation, RNA repositioning, product release, and successive rounds of catalysis, and thereby representing attractive targets for engineering Cid1 catalytic properties.

### 3.5. Other Engineered Residues and Regions

Unlike enzymes that recognize RNA through a discrete RNA-binding domain, Cid1 is thought to bind RNA through a distributed positively charged surface enriched in lysine and arginine residues, including K133, R137, R277, K282, K321, and R323 (Figure 3E). Mutation of several of these basic residues reduces RNA binding and compromises catalytic activity, indicating that RNA recognition is mediated by a broad electrostatic surface rather than a single localized RNA-binding site [62]. Several reported mutations span more than one structural domain and affect catalytic activity to varying degrees, and more details can refer to Table 3.

Beyond individual active-site residues and dynamic structural elements, global engineering of the Cid1 scaffold has also been explored to improve catalytic performance. Terminal truncation generated the engineered PUPdel variant (truncated Cid1 lacking the N- and C-terminal regions; residues 1–12 and 378–405), which exhibited improved incorporation of 3′-O-allyl reversible-terminator nucleotides compared with full-length Cid1 [66]. Simulations suggested that removal of the flexible termini reduces backbone fluctuations and produces a more compact catalytic cleft, providing a possible structural explanation for improved incorporation of modified nucleotides [66]. PUPdel was subsequently used as a scaffold for multi-site engineering, generating variants with substitutions distributed across the hinge regions, CAT domain, CD domain, and distal structural regions. These variants showed improvements in modified-nucleotide incorporation, catalytic efficiency, thermostability, or iterative extension performance [66].

The generatively designed PUPdel2 variant represents a further step toward global optimization of Cid1 properties. Containing 16 substitutions distributed throughout the protein scaffold, PUPdel2 exhibited improved recombinant expression, increased thermostability, and enhanced catalytic efficiency toward selected modified nucleotides compared with PUPdel [66]. Compared with PUPdel, simulations of PUPdel2 indicated reduced fluctuations in two flexible loop regions, suggesting a change in regional conformational stability [66]. The three substitutions in the β-trapdoor region—A297R, S303R, and Q306S (discussed in Section 3.3)—provide a possible explanation for altered substrate access [66]. However, the contributions of individual substitutions and their interactions to the observed gains in expression, thermal stability, and catalytic activity have not been systematically resolved [66]. Overall, PUPdel and PUPdel2 illustrate how scaffold-level engineering can complement changes to nucleotide-recognition residues in improving Cid1 performance [66].

### 3.6. Catalytic Cycle Models

Based on structural and biochemical studies, several complementary catalytic cycle models have been proposed to explain how Cid1 coordinates RNA binding, UTP recognition, nucleotidyl transfer, conformational rearrangement, and product release during iterative uridylation [9,10,61,62,63]. Although these models differ in their interpretation of RNA movement and conformational transitions, they collectively describe a common mechanism in which dynamic structural changes regulate substrate positioning and catalytic progression.

**Figure 4:**
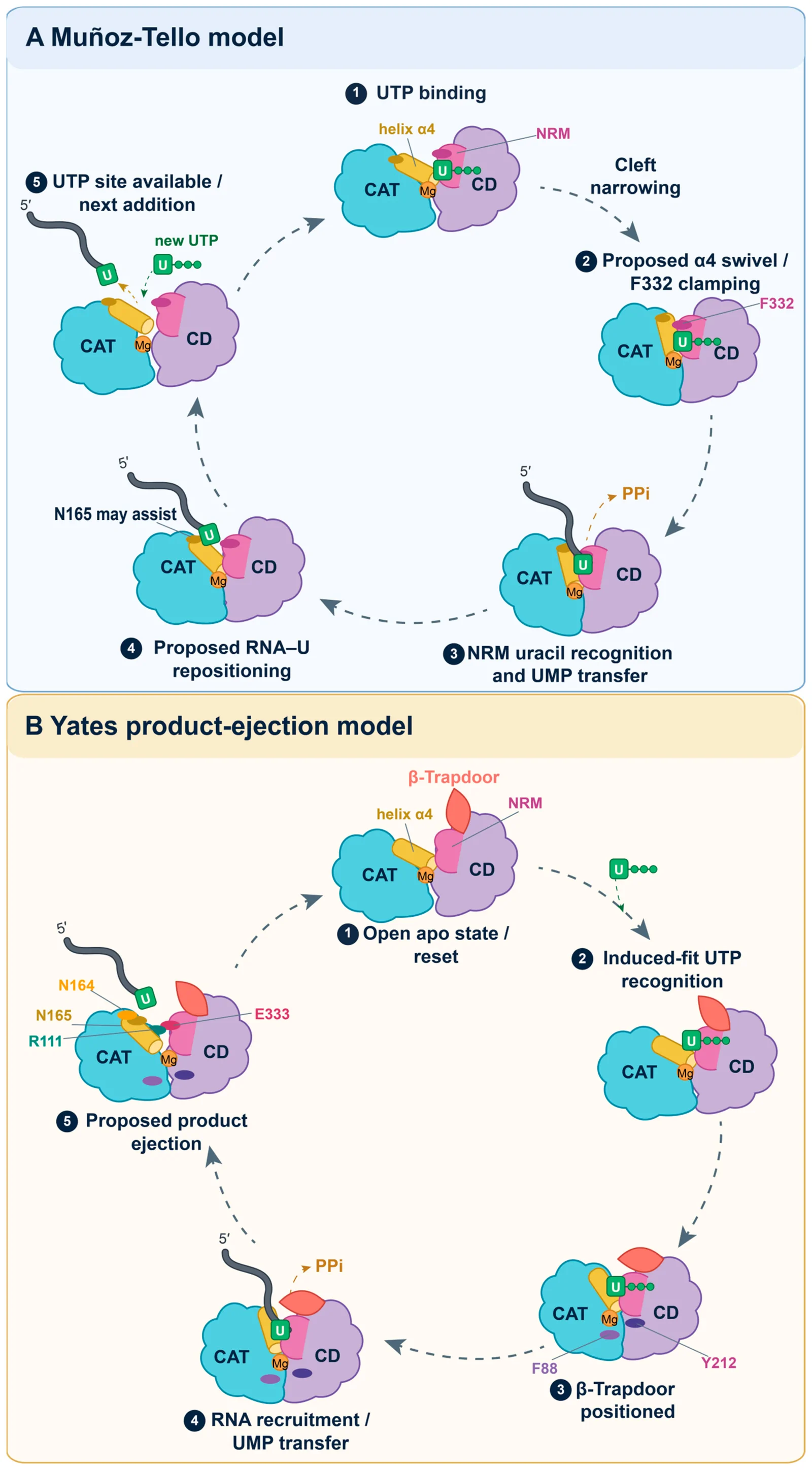
Proposed catalytic cycle models of Cid1. (**A**) Muñoz-Tello model. NTP binding promotes CAT–CD closure and helix 4 rotation, locking F332 and positioning the NRM for uridine recognition. UTP stabilization enables RNA binding and UMP transfer, followed by translocation of the RNA–U product, possibly assisted by N165. This translocation clears the UTP-binding site, allowing a new UTP molecule to bind for another round of UMP addition [10,61]. Adapted and redrawn from Muñoz-Tello et al. [10] under the CC BY-NC 3.0 license. (**B**) Yates product-ejection model. UTP is recognized through induced fit, accompanied by formation of the β-trapdoor, while basic surface patches recruit RNA. UMP transfer and pyrophosphate release are followed by domain rearrangement and ejection of the uridylated RNA product, Cid1 then returns to an open state, and renewed UTP and RNA binding enables another catalytic cycle [63]. Reproduced from Yates et al. [63] under the CC BY 4.0 license.

Muñoz-Tello et al. proposed an early catalytic cycle model based on UTP-bound crystal structures, structural comparisons, and mutational analyses (Figure 4A) [61]. Because Cid1 catalyses template-independent nucleotide addition, substrate recognition and catalysis rely on coordinated local and interdomain rearrangements rather than template-based nucleotide pairing. In this model, UTP binding induces a swivel or ratchet-like movement of the catalytic domain relative to the central domain, centred around helix α4 [61,62,63]. This rearrangement positions F332 and stabilizes the nucleotide-recognition motif, thereby orienting the uracil base for productive catalysis. Following uridyl transfer, N165 near helix α4 is proposed to contribute to repositioning of the RNA 3′ end, allowing another round of UTP binding and nucleotide addition [10,61].

However, subsequent biochemical study indicated that Cid1 activity is predominantly distributive rather than continuously processive on physiological RNA substrates [63]. Cid1 generally adds one or a few uridines to polyadenylated RNA before releasing the substrate, whereas longer U-tails observed under certain in vitro conditions likely arise from repeated cycles of RNA dissociation and rebinding [63]. These findings suggest that iterative translocation within a single enzyme–RNA complex may occur under specific structural conditions but does not represent the only catalytic outcome.

To explain the distributive behaviour of Cid1, Yates et al. proposed a product-ejection model based on additional structural and biochemical analyses (Figure 4B) [62,63]. In this model, UTP binding promotes an induced-fit transition involving H336-mediated nucleotide recognition and Mg^2+^ coordination by the conserved catalytic aspartates [9,62,63]. Closure of the catalytic cleft involves relative movement between the CAT and CD domains, which stabilizes the active conformation and positions the β-trapdoor element to assist in substrate containment. The RNA 3′ end is positioned within the active site through interactions with positively charged regions of the enzyme surface, enabling nucleotidyl transfer.

Following nucleotidyl transfer, the N-terminal catalytic domain undergoes rotational movement relative to the opposing domain around a hinge region centred near residues 164–165 [63]. High-resolution structures captured different closed conformations, including an intermediate state involving approximately 30° rotation and a more extensively closed state displaying approximately 42° rotation [62,63]. This motion markedly reduces the volume of the catalytic cleft and brings the catalytic domain into close proximity with the nucleotide-recognition motif and the bound RNA product [63]. Although the active-site cavity becomes highly constricted in the closed state, a narrow water-filled channel remains accessible and was proposed to provide a possible pathway for pyrophosphate release in the direction opposite to RNA movement [63].

Closure of the catalytic domain brings F88 into close proximity to Y212, with the two aromatic residues forming a hydrophobic constriction within the active site [63]. Comparison with the ApU pseudo-product-bound structure revealed that F88 occupies a position corresponding to the ribose moiety of the terminal uridine in the RNA product mimic rather than the incoming UTP substrate [10,63]. F88 also excludes solvent from part of the closed active pocket. These steric and hydrophobic changes were proposed to destabilize accommodation of the RNA product and thereby contribute to its ejection, although a mechanically directed “outward force” has not been experimentally demonstrated [63].

Additional intramolecular interactions form during catalytic-cleft closure. R111 forms a salt bridge with E333, whereas N164 reorients to interact with E333 [63]. Because E333 and the N164-N165 region are involved in interactions near the RNA 3′ terminus, these newly formed protein–protein contacts were proposed to compete with or weaken enzyme–RNA interactions. The uridylated RNA product could consequently dissociate from the active site, after which Cid1 would return to an open apo-like conformation and become available for another catalytic cycle [10,63].

## 4. Biotechnological Applications of Cid1

**Figure 5:**
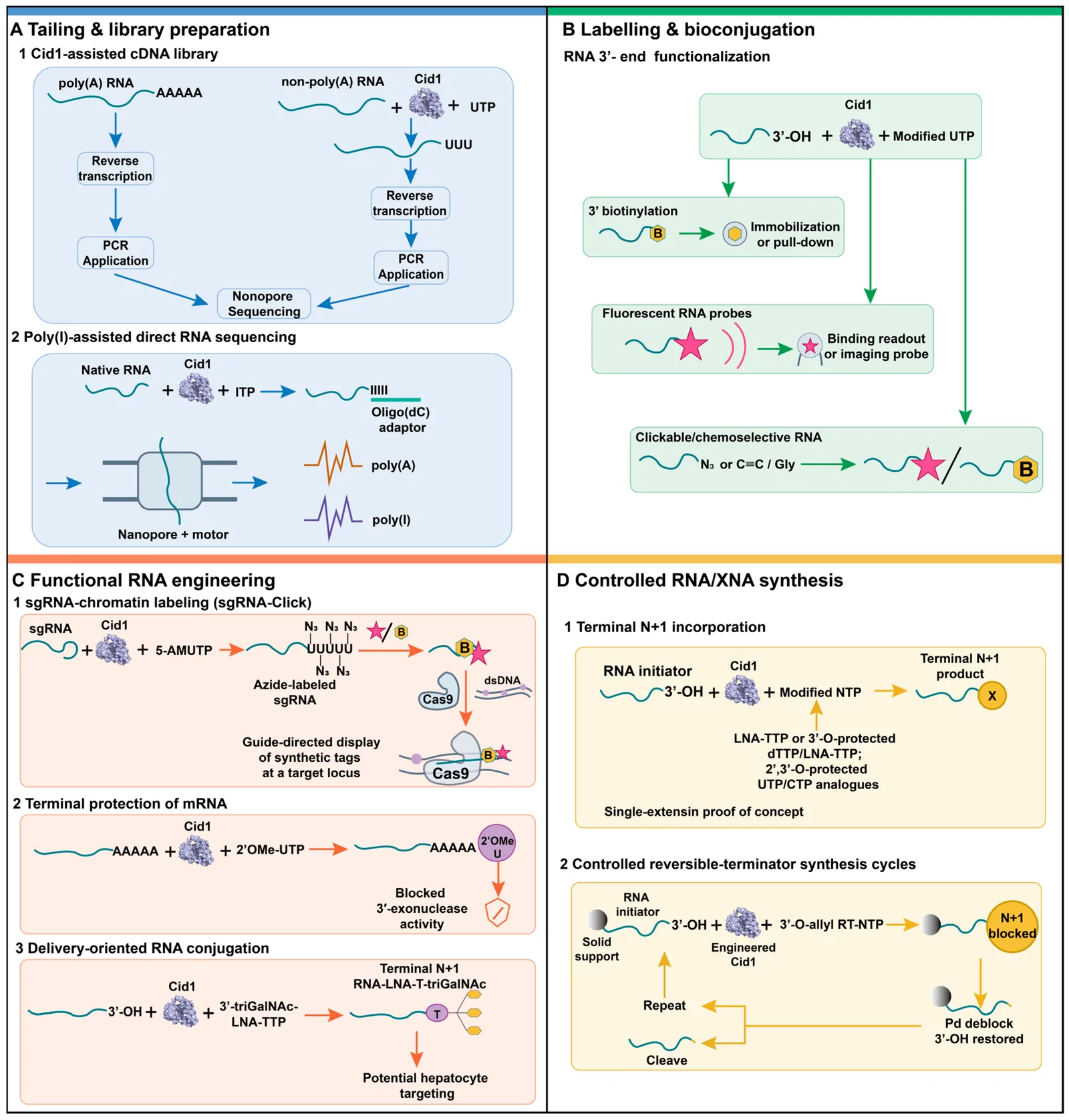
The biotechnological applications of Cid1. (**A**) Tailing and library preparation. (**B**) Labelling and bioconjugation. (**C**) Functional RNA engineering. (**D**) Controlled RNA/XNA synthesis.

Exploiting the natural activity of wild-type Cid1 and the expanded capabilities of engineered variants has enabled four major biotechnological applications (Figure 5), which are discussed in detail in this section. Most applications, however, remain at the research stage, and their broader utility will depend on further validation of delivery, efficacy, and scalability.

### 4.1. RNA Tailing and Sequencing-Library Preparation

Cid1 is commercially available as a research-grade poly(U) polymerase from suppliers such as New England Biolabs (NEB). It can be used for enzymatic tailing of RNA 3′ ends. Artificial nucleotide tails can provide primer-binding or capture sites for applications such as RNA cloning, reverse transcription, and cDNA library preparation [87]. Beyond these conventional applications, Cid1-mediated polyuridylation has been used to generate full-length sequencing libraries from non-polyadenylated RNA (Figure 5A). For example, poly(U) tailing of RNA from HepG2 and HL-7702 cells enabled the detection of full-length splice isoforms and non-polyadenylated transcripts, including growth arrest specific 5 (GAS5) [88].

Cid1-mediated poly(I) tailing has also been explored for nanopore direct RNA sequencing. Poly(I) tails enabled adaptor-mediated capture of both polyadenylated and non-polyadenylated RNAs and produced ionic-current signals that could be distinguished from those generated by native poly(A) tails. This distinction allowed the artificial capture tails to be differentiated from native RNA 3′ ends and enabled the simultaneous analysis of mRNAs, non-poly(A) RNAs and nascent RNAs associated with RNA polymerase II [64]. The poly(I)-tailing approach has also been described in an issued US patent [89], although it has not yet been incorporated into a publicly available commercial nanopore-sequencing kit.

### 4.2. RNA Labelling and Bioconjugation

Cid1 can be used to label the 3′ end of RNA without requiring a specific RNA sequence, providing a flexible approach for introducing affinity, fluorescent, and bioorthogonal groups. One established application is RNA biotinylation using N^6^-(6-aminohexyl)-ATP–biotin, which enables the generation of biotinylated RNA for immobilization, affinity purification, and pull-down experiments (Figure 5B) [77]. This approach has primarily been used as a research method rather than as a dedicated commercial labelling platform.

Cid1 has also been used to incorporate environment-sensitive fluorescent UTP analogues into RNA, generating fluorescent probes for studying RNA–protein interactions and monitoring enzyme–RNA or enzyme–nucleotide interactions through fluorescence-based assays [78]. More recently, azide- and alkyne-containing nucleotides have been incorporated by Cid1 and subsequently coupled to fluorophores through copper-catalysed azide–alkyne cycloaddition (CuAAC) or strain-promoted azide–alkyne cycloaddition (SPAAC). Optimization of the reaction conditions enabled predominantly single-nucleotide incorporation, thereby reducing the heterogeneous products that can arise from extended modified tails [85]. The labelling scope has further been expanded using a glycine-modified UTP, which provides a chemoselective terminal handle for conjugation with affinity tags, fluorescent probes and clickable groups [84]. Collectively, these approaches demonstrate the versatility of Cid1-mediated 3′-end labelling for generating RNA probes, immobilized RNA and late-stage RNA conjugates.

### 4.3. Functional RNA Engineering

Cid1-mediated 3′-end modification can be used not only to label RNA but also to introduce new functions while preserving RNA activity. In the sgRNA-Click platform, Cid1 was used to append azide-modified nucleotides to sgRNAs without disrupting their ability to guide CRISPR–dCas9 to target sites (Figure 5C) [79]. The modified sgRNAs directed the CRISPR complex to selected chromatin regions, where the terminal azide groups were subsequently conjugated to biotin or other synthetic tags. This approach combines programmable genomic targeting with chemical functionalization at the RNA terminus, enabling targeted molecular labelling of chromatin [79].

Cid1 has also been used to modify the 3′ end of mRNA by adding a single 2′-O-methyluridine residue to the end of the poly(A) tail. The terminally modified mRNA showed increased resistance to cellular exonuclease-mediated degradation and produced at least a threefold increase in protein expression in the tested cellular system [90]. This application illustrates a minimally modified mRNA-stabilization strategy in which one terminal nucleotide acts as a protective 3′-end cap. A complementary application installs a targeting ligand at the RNA terminus. Commercial Cid1 incorporated 3′-triGalNAc-LNA-TTP into a natural RNA primer, yielding a terminal N+1 RNA–triGalNAc conjugate (Figure 5C) [83]. This provides an enzymatic route to ligand-bearing RNA, although biological activity, receptor-mediated uptake and therapeutic efficacy of the resulting conjugates were not evaluated [83].

However, these RNA-functionalization strategies remain research-stage applications; therapeutic efficacy, delivery and clinical manufacturing have not yet been established.

### 4.4. Controlled RNA and XNA Synthesis

Cid1 has been investigated as a catalyst for the controlled terminal incorporation of chemically modified RNA and XNA building blocks at the 3′ end of nucleic acids (Figure 5D). In an early application focused on locked nucleic acids (LNAs), poly(U) polymerase was shown to accept several unblocked and 3′-O-protected LNA and DNA nucleotides, producing predominantly terminal N+1 products with approximately 80–95% primer conversion under the reported conditions [81]. Related work extended this approach to 2′,3′-O-protected ribonucleotides, with selected acetal-protected UTP and CTP analogues also supporting efficient single additions [82]. Together, these studies highlight PUP as a candidate catalyst for controlled RNA and XNA synthesis. However, unblocked LNA-TTP terminated further extension and the 3′-O-azidomethyl group of an LNA analogue was lost during or after incorporation [81], whereas acidic deprotection of the isopropylidene-protected RNA product remained partial [82]. Neither study demonstrated a complete iterative extension–deblocking cycle [81,82].

A more advanced approach employed engineered Cid1 variants together with four 3′-O-allyl reversible-terminator NTPs to enable repeated cycles of nucleotide incorporation and deprotection. The platform achieved an average coupling efficiency of approximately 95% and demonstrated ten full liquid-phase synthesis cycles, producing natural and therapeutically relevant modified RNA sequences. The approach was also demonstrated on a controlled-pore-glass support, where N+5 RNA products were synthesized and subsequently released enzymatically (Figure 5D) [65]. This technology has been protected by a Harvard patent family and exclusively licensed to EnPlusOne Biosciences for commercial development [91,92].

Controlled RNA synthesis represents the most advanced, but still proof-of-concept, application of engineered Cid1 [65]. The natural RNA N + 10 product had an isolated purity of 67%, while no more than 50 pmol of a fully modified N + 10 product was recovered from a 200 nmol starting reaction [65]. These results identify cumulative yield loss, inefficient material recovery, incomplete extension, overextension, nucleotide carryover and compatibility between backbone modifications and deblocking chemistry as major barriers. Cid1-based synthesis therefore cannot yet replace phosphoramidite chemistry or in vitro transcription, although its aqueous and template-independent operation may provide a complementary route for preparing selected short or highly modified RNA products under milder conditions [65,93].

## 5. Future Prospects

Although Cid1 has been explored in several RNA research applications, most reported uses remain research-stage demonstrations [64,65,79,85,90]. A useful precedent for advancing Cid1 beyond these demonstrations comes from template-independent DNA synthesis. Like Cid1, terminal deoxynucleotidyl transferase (TdT) is a template-independent polymerase that can add nucleotides to a nucleic acid 3′ end without requiring a complementary template. However, whereas Cid1 naturally functions as an RNA terminal nucleotidyltransferase, TdT has been more extensively developed as a platform for controlled enzymatic DNA synthesis. This precedent suggests that converting a template-independent polymerase into a practical synthesis platform requires coordinated optimization of the enzyme, nucleotide design, reaction cycling, product handling, solid-phase processing, and automation [93,94,95,96].

For Cid1, key priorities include reducing disparities in the incorporation efficiency of natural and modified nucleotide substrates, reducing sensitivity to the primer-terminal sequence and RNA secondary structure, increasing tolerance of sugar and backbone modifications, and limiting incomplete and overextended products [65,66]. Beyond demonstrating acceptance of additional nucleotide substrates, a central goal is to achieve reliable synthesis of longer, defined RNA sequences with high cumulative yield, low insertion and deletion error rates, and compatibility with scalable solid-phase, flow-based, or automated workflows [65,93].

## 6. Conclusions

Cid1 provides a well-characterized model for understanding how template-independent nucleotide addition is governed by nucleotide recognition, RNA 3′-end positioning, and conformational dynamics. The studies reviewed here indicate that its activity and substrate preferences arise from coordinated interactions among the catalytic cleft, nucleotide-recognition motif, interdomain hinges, and β-trapdoor, rather than from a single structural determinant. These insights have guided the engineering of Cid1 variants with altered nucleotide preferences and improved incorporation of selected chemically modified substrates, linking mechanistic understanding to the development of new RNA tools. Applications in RNA sequencing, terminal labelling, functional RNA modification, and controlled oligonucleotide synthesis demonstrate the versatility of wild-type and engineered Cid1. However, the acceptance of individual nucleotide substrates does not necessarily translate into efficient, reproducible modification or multicycle synthesis. Uneven substrate incorporation, product heterogeneity, and cumulative yield losses remain important constraints. Further progress will depend on integrating enzyme engineering with nucleotide chemistry and process optimization, supported by validation across diverse RNA substrates. Cid1 therefore offers both a model for RNA terminal nucleotidyl transfer and a foundation for developing more versatile and controlled methods of RNA synthesis and 3′-end functionalization.

## Figures and Tables

**Table 1:** Representative Cid1-family and functionally related terminal nucleotidyltransferases in eukaryotes.

Species; Protein (UniProt)	Class; Substate → Tail Product	EC Number	Length (Amino Acid)	Main Biological Role	Pfam Domains	Cofactor; PDB ID	Key References
*S. pombe*; Cid1 (O13833)	PUP/TUTase; UTP → oligo/poly(U) tails	2.7.7.52	405	Uridylates shortened cytoplasmic mRNA poly(A) tails, enhances decapping and favours 5′ → 3′ mRNA decay; limits excessive deadenylation and 3′ → 5′ decay.	PF01909/PF22600 + PF03828	Mg^2+^/Mn^2+^; PDB 4E7X; 4E80; 4E8F; 4EP7; 4FH3; 4FH5; 4FHP; 4FHV; 4FHW; 4FHX; 4FHY; 4NKT; 4NKU; 4UD4; 4UD5	[3,6,9,10]
*S. pombe*; Cid16 (O13798)	PUP/TUTase; UTP → mono/oligo(U) tails	2.7.7.52 *	1202	Uridylates non-polyadenylated or deadenylated RNA and promotes Rrp6-mediated turnover of Ago1-bound sRNAs.	PF01909/PF22600	-; -	[3,11,12]
*C. elegans*; CDE-1/PUP-1/CID-1 (Q09409)	Cid1-related PUP/TUTase; UTP → mono/oligo(U) tails	2.7.7.52 *	1425	Uridylates CSR-1 22G RNAs and rRNA; supports germline and chromosome fidelity.	PF01909/PF22600 + PF03828.	-; -	[13,14,15]
*C. elegans*; PUP-2 (Q09408)	Cid1-related PUP/TUTase; UTP → mono/oligo(U) tails	2.7.7.52 *	508	LIN-28-dependent pre-let-7 regulation and U-tailing of selected small RNAs.	PF01909/PF22600 + PF03828	-; -	[13,16,17]
*C. elegans*; PUP-3 (P91361)	Cid1-related PUP/TUTase; UTP → oligo(U) tails	2.7.7.52 *	482	Major U-tail contributor for non-adenylated mRNAs and selected small RNAs.	PF01909/PF22600 + PF03828	-; -	[13,17]
*C. elegans*; PUP-4/F43E2.1 (O02094)	Putative/atypical PUP/TUTase; UTP → U-tails	2.7.7.52 *	467	Modulates U-tailing of discrete small-RNA subsets; modest germline contribution.	PF01909/PF22600	-; -	[13,17]
*H. sapiens*; TUT4/ZCCHC11/TENT3A (Q5TAX3)	TUTase; UTP → mono/oligo(U) tails	2.7.7.52	1644	Pre-let-7, miRNA and mRNA U-tailing; RNA decay control and LINE-1 restriction.	PF01909/PF22600 + PF03828 + PF19088 + PF00098	Mg^2+^/Mn^2+^/Zn^2+^; PDB 6IW6; 8OST; 9W4R; 9W4S	[18,19,20,21,22,23]
*H. sapiens*; TUT7/ZCCHC6/TENT3B (Q5VYS8)	PUP/TUTase; UTP → mono/oligo(U) tails	2.7.7.52	1,495	Overlaps with TUT4 in pre-let-7, miRNA/mRNA U-tailing and RNA turnover.	PF01909/PF22600 + PF03828 + PF19088 + PF16631 + PF00098	Mg^2+^/Mn^2+^/Zn^2+^; PDB 5W0B; 5W0M; 5W0N; 5W0O; 8OEF; 8OPP; 8OPS; 8OPT	[20,21,22,23,24]
*D. melanogaster*; Tailor (Q9VI58)	PUP/TUTase; UTP → mono/oligo(U)	2.7.7.52	560	Uridylates 3′-G-ended mirtrons or RNAs and suppresses mirtron biogenesis.	PF01909/PF22600	Mg^2+^; PDB 5Z4A; 5Z4C; 5Z4D; 5Z4J; 5Z4M; 6I0S; 6I0T; 6I0U; 6I0V	[25,26,27,28]
*A. thaliana*; HESO1 (Q5XET5)	PUP/TUTase; UTP → oligo/poly(U) tails	2.7.7.52	511	Uridylates unmethylated small RNAs and AGO1-cleaved RNA fragments for turnover.	PF01909/PF22600	-; -	[29,30,31,32,33]
*A. thaliana*; URT1 (O64642)	PUP/TUTase; UTP → mono/oligo(U) tails	2.7.7.52	764	Prevents mRNA 3′ trimming and aberrant siRNA formation; cooperates with HESO1 on miRNAs and RISC 5′ fragments.	PF01909/PF22600 + PF03828	Mg^2+^/Mn^2+^; PDB 6L3F; 6L8K; 7XS4	[31,34,35,36,37]
*S. pombe*; Cid11 (O74326)	Cid1-family ncPAP; ATP → oligo(A) tails, endogenous product unknown	2.7.7.19	478	RNA 3′-end A-addition; endogenous substrates and physiological functions remain unclear.	PF01909/PF22600 + PF03828	Mg^2+^/Mn^2+^; -	[8,38]
*S. pombe*; Cid12 (O74518)	Cid1-family ncPAP; RDRC subunit; ATP → mono(A) tails	2.7.7.19	336	RDRC-dependent siRNA biogenesis and heterochromatin silencing; chromosome segregation and DNA replication checkpoint control.	PF01909/PF22600 + PF03828	Mg^2+^/Mn^2+^; -	[39,40,41]
*S. pombe*; Cid13 (Q9UT49)	Cid1-family ncPAP; ATP → oligo/poly(A) tails	2.7.7.19	578	Polyadenylates and stabilizes suc22 mRNA during replication stress.	PF01909/PF22600 + PF03828	Mg^2+^/Mn^2+^; -	[8,42]
*S. pombe*; Cid14 (Q9UTN3)	Nuclear/TRAMP-associated ncPAP; ATP → oligo(A) tails	2.7.7.19	684	Nuclear RNA surveillance, rRNA quality control, heterochromatin and chromosome stability.	PF01909/PF22600 + PF03828	Mg^2+^/Mn^2+^; -	[43,44,45,46]
*H. sapiens*; TENT2/GLD2/PAPD4 (Q6PIY7)	ncPAP; ATP → mono/poly(A) tails	2.7.7.19	484	stabilizes selected miRNAs and promotes QKI-7-directed mRNA translation.	PF01909/PF22600 + PF03828	Mg^2+^/Mn^2+^; -	[47,48,49,50,51]
*H. sapiens*; TENT4A/PAPD7 (Q5XG87)	ncPAP; ATP/GTP → mixed A/G tails	2.7.7.19	792	Mixed A/G tails stabilize selected cellular and viral RNAs; short oligo(A) contributes to nuclear RNA surveillance.	PF01909/PF22600 + PF03828.	Mg^2+^/Mn^2+^; -	[47,51,52,53,54,55]
*H. sapiens*; TENT4B/PAPD5 (Q8NDF8)	ncPAP; ATP/GTP → mixed A/G tails	2.7.7.19	572	Mixed A/G tailing-mediated RNA stabilization and oligo(A)-dependent nuclear RNA surveillance.	PF01909/PF22600 + PF03828.	Mg^2+^/Mn^2+^; -	[47,51,52,53,54,56,57]

ncPAP, non-canonical poly(A) polymerase; PUP, poly(U) polymerase; TENT, terminal nucleotidyltransferase; TUTase, terminal uridylyltransferase; RDRC, RNA-directed RNA polymerase complex; TRAMP, nuclear RNA-surveillance polyadenylation complex; PF01909: Nucleotidyltransferase domain, older broad annotation; currently represented by PF22600; PF22600: Poly(A) RNA polymerase, mitochondrial-like, central palm domain; PF03828: Cid1 family poly A polymerase; PF19088: TUTase nucleotidyltransferase domain; PF00098: Zinc knuckle; PF16631: Unstructured region 4 on terminal uridylyltransferase 7; * Marked: putative EC number; actual EC is not curated; -: not available.

**Table 2:** Summary of natural and modified nucleotide utilization by Cid1 and its variants for RNA 3′-end extension.

No.	Substrate Name	Molecular Formula (Free Acid)	Extension Products	Enzyme/Variant	References
1	UTP	C9H15N2O15P3	Multiple nucleotide additions	Cid1 (31–377)/WT Cid1 (1–405)	[9,65]
2	ATP	C10H16N5O13P3	Multiple nucleotide additions	Cid1 (31–377)/WT Cid1 (1–405)	[9,65]
3	CTP	C9H16N3O14P3	Conditional—absent in Lunde assay; positive extension in later assays	Cid1 (31–377)/WT Cid1 (1–405)	[9,65]
4	GTP	C10H16N5O14P3	Conditional—weak in Lunde assay; positive extension in later assays	Cid1 (31–377)/WT Cid1 (1–405)	[9,65]
5	ITP	C10H15N4O14P3	Multiple nucleotide additions	Commercial NEB PUP	[64]
6	5-methyl-UTP	C10H17N2O15P3	Multiple nucleotide additions	Commercial NEB PUP	[64]
7	4-thio-UTP	C9H15N2O14P3S	Multiple nucleotide additions	Commercial NEB PUP	[64]
8	N^6^-(6-aminohexyl)-ATP–biotin	C26H43N8O15P3S	Multiple nucleotide additions	Cid1 (32–405)	[77]
9	BFUTP [5-(benzofuran-2-yl)-UTP]	C17H19N2O16P3	Multiple nucleotide additions	Recombinant SpCid1	[78]
10	AMUTP (5-azidomethyl-UTP)	C10H16N5O15P3	Multiple nucleotide additions	Recombinant SpCid1	[79]
11	SeUTP [5-(selenophen-2-yl)-UTP]	C13H17N2O15P3Se	One/few additions; limited extension	Recombinant SpCid1	[78]
12	APUTP [5-(3-azidopropyl)-UTP]	C12H20N5O15P3	One/few additions; limited extension	Recombinant SpCid1	[79]
13	ATUTP (C5-alkynyl, tetraethylene-glycol–azide UTP)	C20H32N5O19P3	One/few additions; limited extension	Recombinant SpCid1	[79]
14	2′-O-methyl-UTP	C10H17N2O15P3	One/few additions; limited extension	Commercial NEB PUP	[64]
15–18	2′-Azido-2′-deoxy-NTPs (A/U/G/C)	A: C10H15N8O12P3 U: C9H14N5O14P3 G: C10H15N8O13P3 C: C9H15N6O13P3	Low/variable conversion; mixed product lengths	Commercial NEB PUP	[80]
19	LNA-TTP	C11H17N2O15P3	Single addition (+1)	Commercial PUP	[81]
20	3′-O-allyl-dTTP	C13H21N2O14P3	Single addition (+1)	Commercial PUP	[81]
21	3′-O-azidomethyl-dTTP	C11H18N5O14P3	Single addition (+1)	Commercial PUP	[81]
22	3′-O-allyl-LNA-TTP	C14H21N2O15P3	Single addition (+1)	Commercial PUP	[81]
23	3′-O-methyl-LNA-TTP	C12H19N2O15P3	Single addition (+1)	Commercial PUP	[81]
24	3′-O-azidomethyl-LNA-TTP	C12H18N5O15P3	Single addition (+1)	Commercial PUP	[81]
25	2′,3′-O-isopropylidene-UTP (6a)	C12H19N2O15P3	Predominantly +1; high conversion after optimization	Commercial PUP	[82]
26	2′,3′-O-cyclohexylidene-UTP (6b)	C15H23N2O15P3	Predominantly +1 after optimization	Commercial PUP	[82]
27	2′,3′-O-benzylidene-UTP (6c)	C16H19N2O15P3	Predominantly +1 after optimization	Commercial PUP	[82]
28	2′,3′-O-(2-methoxycarbonyl) ethylidene-UTP (6d)	C13H19N2O17P3	Predominantly +1 (~90% under reported conditions)	Commercial PUP	[82]
29	2′,3′-O-isopropylidene-CTP (12a)	C12H20N3O14P3	Efficient +1; minor (<5%) N+2	Commercial PUP	[82]
30	2′,3′-O-isopropylidene-GTP (12b)	C13H20N5O14P3	Moderate +1 (~50% at 50 µM)	Commercial PUP	[82]
31	2′,3′-O-cyclohexylidene-ATP (13)	C16H24N5O13P3	Weak +1	Commercial PUP	[82]
32	3′-triGalNAc-LNA-TTP (4)	C77H128N13O47P3	Terminal +1 on natural RNA P2	Commercial NEB PUP	[83]
33	GUTP (glycine-modified UTP)	C12H21N4O16P3	Transfer reported—length category unresolved	Cid1 (40–377)	[84]
34	2′,3′-O-isopropylidene-ATP (12c)	C13H20N5O13P3	Mixed extension—intact protected transfer unresolved	Commercial PUP	[82]
35	2′,3′-O-isopropylidene-ITP (12d)	C13H19N4O14P3	Mixed extension—intact protected transfer unresolved	Commercial PUP	[82]
36	2′,3′-bis-O-acetyl-UTP (4a)	C13H19N2O17P3	Mixed extension—intact protected transfer unresolved	Commercial PUP	[82]
37	2′,3′-bis-O-benzoyl-UTP (4b)	C23H23N2O17P3	Mixed extension—intact protected transfer unresolved	Commercial PUP	[82]
38	2′,3′-O-trinitrophenyl-UTP (TNP-UTP; 6e)	C15H16N5O21P3	Mixed extension—intact protected transfer unresolved	Commercial PUP	[82]
39–42	2′-Fluoro-2′-deoxy-NTPs (A/U/G/C)	A: C10H15FN5O12P3 U: C9H14FN2O14P3 G: C10H15FN5O13P3 C: C9H15FN3O13P3	Multiple nucleotide additions	H336R; H336R/N171A/T172S	[65]
14; 43–45	2′-O-methyl-NTPs (A/U/G/C)	A: C11H18N5O13P3 U: C10H17N2O15P3 G: C11H18N5O14P3 C: C10H18N3O14P3	Multiple nucleotide additions	H336R; H336R/N171A/T172S	[65]
46–49	α-PS-NTP (A/U/G/C)	A: C10H16N5O12P3S U: C9H15N2O14P3S G: C10H16N5O13P3S C: C9H16N3O13P3S	Multiple nucleotide additions	H336R; H336R/N171A/T172S	[65]
50	N^6^-propargyl-ATP	C13H18N5O13P3	Long tails	H336R; H336R/N171A/T172S	[65]
51	2-ethynyl-ATP	C12H16N5O13P3	Long tails	H336R; H336R/N171A/T172S	[65]
52–55	dNTPs (dATP/dTTP/dGTP/dCTP)	A: C10H16N5O12P3 T: C10H17N2O14P3 G: C10H16N5O13P3 C: C9H16N3O13P3	Limited extension	H336R; H336R/N171A/T172S	[65]
56–59	3′-O-Allyl-NTPs (A/U/G/C)	A: C13H20N5O13P3 U: C12H19N2O15P3 G: C13H20N5O14P3 C: C12H20N3O14P3	+1; iterative cycles demonstrated	H336R; H336R/N171A/T172S; Yang truncated/engineered variants	[65,66]
60–63	3′-O-Allyl-2′-fluoro-2′-deoxy-NTPs (A/U/G/C)	A: C13H19FN5O12P3 U: C12H18FN2O14P3 G: C13H19FN5O13P3 C: C12H19FN3O13P3	+1, deblocking, then +2	H336R	[65]
64–67	3′-O-Allyl-2′-O-methyl-NTPs (A/U/G/C)	A: C14H22N5O13P3 U: C13H21N2O15P3 G: C14H22N5O14P3 C: C13H22N3O14P3	+1, deblocking, then +2	H336R	[65]
68–71	3′-O-Allyl-NTP-α-PS (A/U/G/C)	A: C13H20N5O12P3S U: C12H19N2O14P3S G: C13H20N5O13P3S C: C12H20N3O13P3S	+1; PS damaged during Pd/TPPTS deblocking	H336R	[65]
72–75	3′-O-Propargyl-NTPs (A/U/G/C)	A: C13H18N5O13P3 U: C12H17N2O15P3 G: C13H18N5O14P3 C: C12H18N3O14P3	Single addition; terminal alkyne retained	H336R; H336R/N171A/T172S	[65]
21; 76–78	3′-O-Azidomethyl-dNTPs (A/T/G/C)	A: C11H17N8O12P3 T: C11H18N5O14P3 G: C11H17N8O13P3 C: C10H17N6O13P3	Single addition (+1)	H336R	[65]
79–81	3′-O-Azidomethyl-NTP-α-PS (A/U/C)	A: C11H17N8O12P3S U: C10H16N5O14P3S C: C10H17N6O13P3S	+1; deblocking verified	H336R	[65]
82	3′-O-Allyl-ITP	C13H19N4O14P3	+1; EndoV junction application	H336R	[65]

The chemical structures for substrates 1–82 listed in this table are shown in the Supplementary Material.

**Table 3:** Reported mutations of Cid1 and their effects on poly(U) polymerase activity.

Mutation/Variant	Specific Structural Domain	Functional Impact as Reported	Reference
F88D	CAT domain (residues 56–165)	Reduced or defective PUP activity; implicated in structural rearrangement/product release.	[63]
D101A/D103A	CAT domain	Abolished catalytic activity but did not abolish RNA binding	[62]
R139A	CAT domain	Reported as similar to WT in a product-bound study, but the earlier assay indicated reduced PUP activity	[10,61]
K144A	CAT-domain	Reduced association with A15 RNA but not U15 RNA; PUP activity comparable to WT, while PAP activity was drastically reduced and became more distributive.	[10]
D160A	CAT domain	Catalytically inactive	[9,10]
C63I-PUPdel	CAT domain; C63-PUPdel corresponds to full-length C75	Improved thermal stability relative to PUPdel	[66]
D97P-PUPdel	CAT domain; D97-PUPdel corresponds to full-length D109	Improved thermal stability relative to PUPdel	[66]
I128V-PUPdel	CAT domain; I128-PUPdel corresponds to full-length I140	Slightly improved elongation with unmodified NTP compared with PUPdel.	[66]
K132R-PUPdel	CAT domain; K132-PUPdel corresponds to full-length K144	Modelled to strengthen electrostatic attraction; measured thermal stability was comparable to PUPdel	[66]
Y205D; Y205F; Y205V	CD domain (residues 40–55 and 166–377)	All three substitutions decreased PUP activity, with a particularly marked reduction reported for Y205D.	[61]
D330A	CD domain; NRM (residues 327–339)	Reduced but did not abolish PUP activity; D330 participates in the H336-associated nucleotide recognition network.	[62]
F332L; F332A; F332D	CD domain; NRM	F332L retained PUP activity; F332A reduced RNA binding and PUP activity; F332D almost abolished detectable PUP activity.	[10,61]
E333A	CD domain; NRM	Greatly diminished PUP activity	[62]
H336A; H336N; H336L; H336R	CD domain; NRM	H336A showed poly(A) polymerase (PAP) activity in one assay, although another study reported reduced activity with both ATP and UTP. H336N decreased PUP activity but increased ATP incorporation; H336L reduced activity with both nucleotides while retaining a preference for UTP. H336R was used to broaden incorporation of 3′-O-allyl reversible-terminator NTPs.	[9,61,62,65]
H336R/N171A/T172S	CD domain; NRM;	Engineered triple mutant used for incorporation of 3′-O-allyl reversible terminator NTPs; broadened substrate compatibility relative to wild-type Cid1/PUP.	[65]
N159F-, N159I-, N159Q-, and N159M-PUPdel	CD domain; N159-PUPdel corresponds to full-length N171	All four retained 3′-O-allyl-NTP incorporation in the initial screen. N159F (MT2) was characterized further: it achieved greater than 95% incorporation under the reported assay conditions and increased catalytic efficiency for 3′-O-allyl-UTP and -CTP relative to PUPdel.	[66]
L223Q-PUPdel	CD domain; L223-PUPdel corresponds to full-length L235	Proposed to form a new hydrogen bond with P319 and improve local stability.	[66]
Δ310–322	β-trapdoor (residues 310–322); CD domain	RNA binding not strongly affected, but catalysis became more distributive, especially with PAP activity.	[10]
I45P(MT1)-PUPdel	The first CAT–CD hinge (56–58); I45- PUPdel corresponds to full-length I57	Increased catalytic efficiency toward 3′-O-allyl-CTP by about 4-fold; improved thermostability.	[66]
N164P	The second CAT–CD hinge (164–165)	Predominantly monouridylation;	[63]
N164P/N165P	The second CAT–CD hinge	Defective PUP activity; limiting hinge flexibility impairs domain motion and product release.	[63]
N165A	The second CAT–CD hinge	monouridylation; RNA binding is not strongly impaired.	[10,63]
N165D	The second CAT–CD hinge	With 15-nt RNA primer, could add approximately 3–4 U or up to ~20 A; nearly unable to use ApU substrate; reduced catalytic activities toward ATP and UTP.	[10]
K133A/R137A	Positively charged surface; CAT domain	Reduced RNA binding	[62]
R277A/K282A	Positively charged surface; CD domain	Reduced RNA binding	[62]
K321A/R323A	Positively charged surface; CD domain; β-trapdoor	Reduced RNA binding	[62]
R277A/K321A/R323A	Positively charged surface; CD domain; β-trapdoor	Reduced RNA binding	[62]
K282A/K321A/R323A	Positively charged surface; CD domain; β-trapdoor	Reduced RNA binding	[62]
K144A/R277A/K282A	Positively charged surface; CAT + CD domain	Defective PUP activity; RNA-binding patch mutations.	[62]
K133A/R137A/R277A	Positively charged surface; CAT + CD domain	Reduced RNA binding	[62]
K133A/R137A/K282A	Positively charged surface; CAT + CD domain	Reduced RNA binding.	[62]
K133A/R137A/R277A/K282A	Positively charged surface; CAT + CD domain	Reduced RNA binding	[62]
K133A/R137A/K321A/R323A	Positively charged surface; CAT + CD domain	Reduced RNA binding	[62]
R277A/K282A/K321A/R323A	Positively charged surface; CD domain	Reduced RNA binding	[62]
D160A/H336N	CAT + CD domain	Catalytically inactive	[9]
PUPdel	N-terminal Δ1–12 + C-terminal Δ378–405	N- and C-terminal truncation; increased conformational stability and improved incorporation of 3′-O-allyl-NTPs.	[66]
I45L/L223Q(MT21)-PUPdel	CAT + CD domain; I45/L223-PUPdel corresponds to full-length I57/L235	Improved catalytic efficiency toward 3′-O-allyl-GTP and 3′-O-allyl-CTP by about 2-fold.	[66]
I45P/I128V/L223Q(MT31)-PUPdel	CAT + CD domain; I45/I128/L223-PUPdel corresponds to full-length I57/I140/L235	Multi-mutant evaluated for combined activity/stability.	[66]
I45P/L223Q/P273Y(MT32)-PUPdel	CAT + CD domain; I45/L223/P273-PUPdel corresponds to full-length I57/L235/P285	Improved incorporation of some 3′-O-allyl-NTPs, especially moderate improvement for CTP/GTP analogs.	[66]
I45P/I128V/S165T/L223Q(MT41)-PUPdel	CAT + CD domain; I45/I128/S165/L223-PUPdel corresponds to full-length I57/I140/S177/L235	Improved 3′-O-allyl-GTP/CTP catalytic efficiency by about 2-fold; supported iterative n+1 incorporation, deblocking, and n+2 extension.	[66]
I45P/L223Q/K270Q/P273Y(MT42)-PUPdel	CAT-CD hinge + CD domain; I45/L223/K270/P273-PUPdel corresponds to full-length I57/L235/K282/P285	Increased thermostability; Tm about 6.4 °C higher than PUPdel.	[66]
PUPdel2	CAT + CD + NRM + β-trapdoor; P26S/A55N/D58E/D70N/A71S/C147I/Y208H/F219L/S225R/S254E/R282K/A297R/S303R/Q306S/T329S/S360K corresponds to full-length P38S/A67N/D70E/D82N/A83S/C159I/Y220H/F231L/S237R/S266E/R294K/A309R/S315R/Q318S/T341S/S372K	ESM3/generative-design variant with 16 substitutions; increased Tm by ~3.4 °C, increased expression by ~3.7-fold, and improved 3′-O-allyl-UTP catalytic efficiency up to ~5.4-fold; A297R/S303R/Q306S within PUPdel2 altered local electrostatics and β-trapdoor flexibility; proposed to create a dynamic “attractive gateway” for substrate entry.	[66]

## Data Availability

No new data were created or analyzed in this study. Data sharing is not applicable to this article.

## Supplementary Materials

The following supporting information can be downloaded at: https://www.mdpi.com/article/10.3390/biom16101535/s1, Figure S1: Chemical structures of nucleotide substrates accepted by Cid1 and its engineered variants; Table S1: Additional information and sequences used to build phylogenetic tree and domains prediction.
