# Supplementary material for: Structure–Function Relationships of Cid1 Poly(U) Polymerase: Catalysis, Substrate Recognition, Engineering, and Applications
Source: Biomolecules. 2026 Oct 21;16(10):1535. doi: 10.3390/biom16101535 (PMC13702519; doi:10.3390/biom16101535)

Figure S1: Chemical structures of nucleotide substrates accepted by Cid1 and its engineered variants

This supplement presents 82 individual nucleotide chemical structures, designated S001–S082. These structures identifiers correspond to those listed in Table 2 of the main text. Compound numbers in parentheses correspond to the numbering used in the respective source publications. All chemical structures were prepared using ChemDraw software (version 23.1.1; Revvity Signals Software, Inc., Waltham, MA, USA).

S001 UTP

Molecular formula (neutral free acid): C9H15N2O15P3

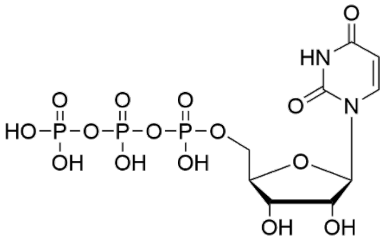

S002 ATP

Molecular formula (neutral free acid): C10H16N5O13P3

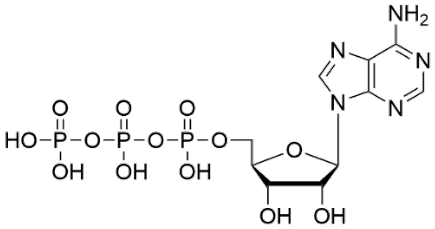

S003 CTP

Molecular formula (neutral free acid): C9H16N3O14P3

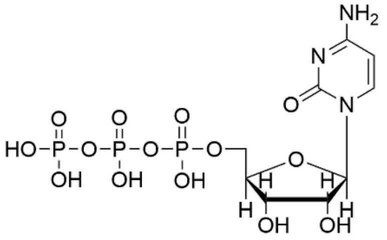

S004 GTP

Molecular formula (neutral free acid): C10H16N5O14P3

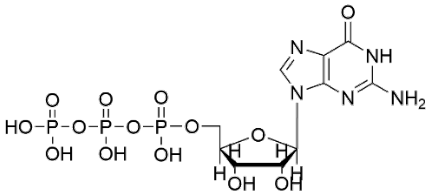

S005 ITP

Molecular formula (neutral free acid): C10H15N4O14P3

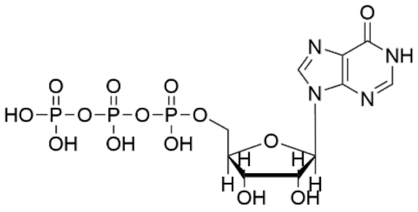

S006 5-methyl-UTP

Molecular formula (neutral free acid): C10H17N2O15P3

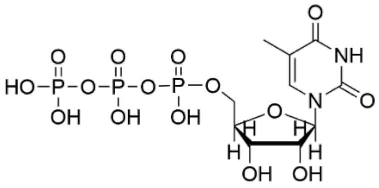

S007 4-thio-UTP

Molecular formula (neutral free acid): C9H15N2O14P3S

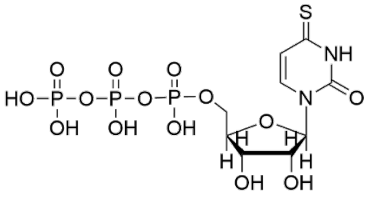

S008 N[6]-(6-aminohexyl)-ATP–biotin

Molecular formula (neutral free acid): C26H43N8O15P3S

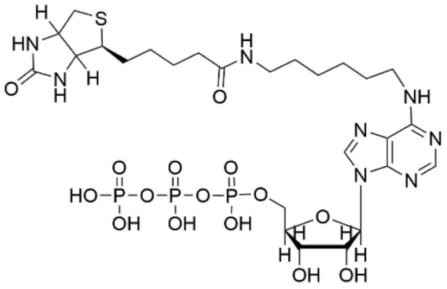

S009 BFUTP [5-(benzofuran-2-yl)-UTP]

Molecular formula (neutral free acid): C17H19N2O16P3

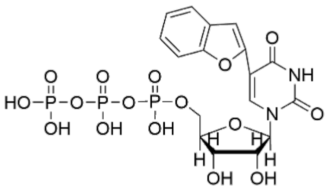

S010 AMUTP (5-azidomethyl-UTP)

Molecular formula (neutral free acid): C10H16N5O15P3

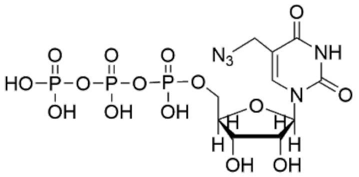

S011 SeUTP [5-(selenophen-2-yl)-UTP]

Molecular formula (neutral free acid): C13H17N2O15P3Se

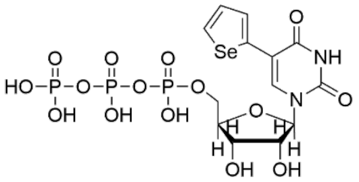

S012 APUTP [5-(3-azidopropyl)-UTP]

Molecular formula (neutral free acid): C12H20N5O15P3

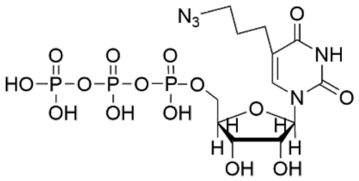

S013 ATUTP (C5-alkynyl, tetraethylene-glycol–azide UTP)

Molecular formula (neutral free acid): C20H32N5O19P3

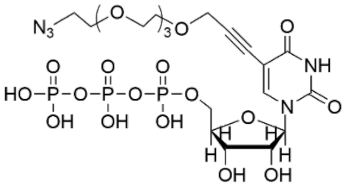

S014 2′-O-methyl-UTP

Molecular formula (neutral free acid): C10H17N2O15P3

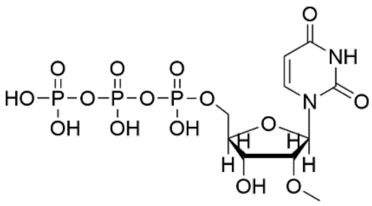

S015 2′-azido-2′-deoxy-ATP

Molecular formula (neutral free acid): C10H15N8O12P3

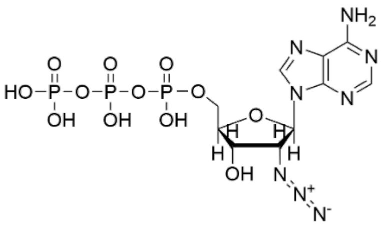

S016 2′-azido-2′-deoxy-UTP

Molecular formula (neutral free acid): C9H14N5O14P3

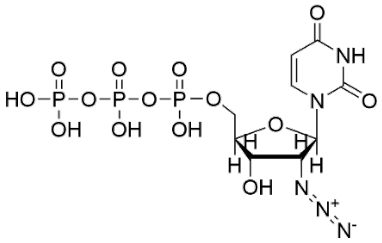

S017 2′-azido-2′-deoxy-GTP

Molecular formula (neutral free acid): C10H15N8O13P3

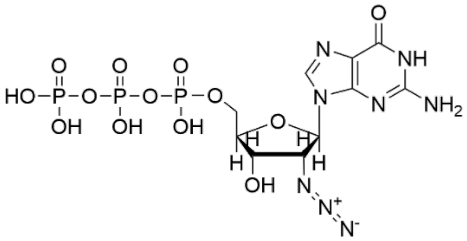

S018 2′-azido-2′-deoxy-CTP

Molecular formula (neutral free acid): C9H15N6O13P3

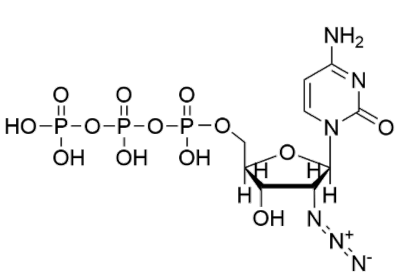

S019 LNA-TTP

Molecular formula (neutral free acid): C11H17N2O15P3

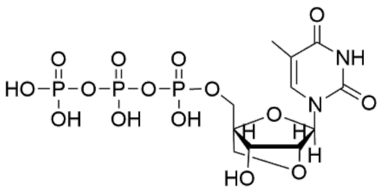

S020 3′-O-allyl-dTTP

Molecular formula (neutral free acid): C13H21N2O14P3

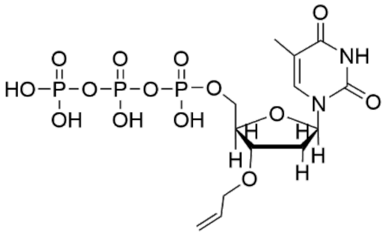

S021 3′-O-azidomethyl-dTTP

Molecular formula (neutral free acid): C11H18N5O14P3

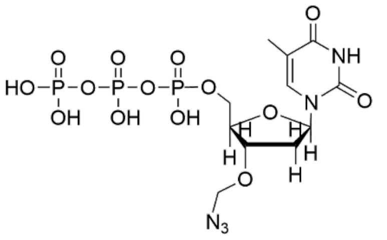

S022 3′-O-allyl-LNA-TTP

Molecular formula (neutral free acid): C14H21N2O15P3

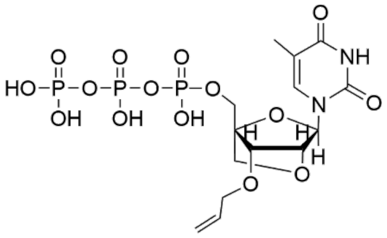

S023 3′-O-methyl-LNA-TTP

Molecular formula (neutral free acid): C12H19N2O15P3

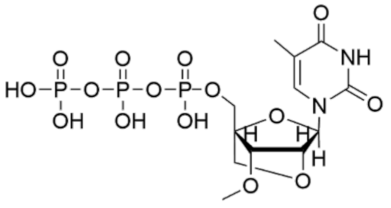

S024 3′-O-azidomethyl-LNA-TTP

Molecular formula (neutral free acid): C12H18N5O15P3

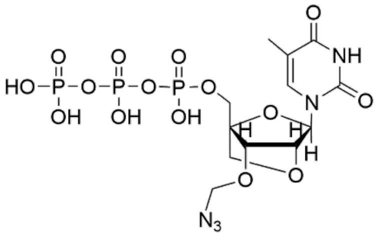

S025 2′,3′-O-isopropylidene-UTP (6a)

Molecular formula (neutral free acid): C12H19N2O15P3

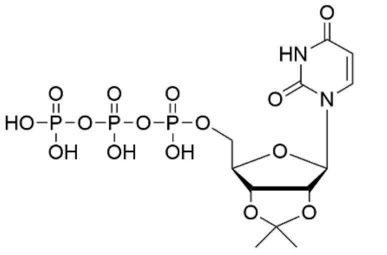

S026 2′,3′-O-cyclohexylidene-UTP (6b)

Molecular formula (neutral free acid): C15H23N2O15P3

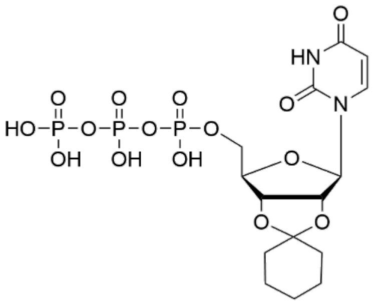

S027 2′,3′-O-benzylidene-UTP (6c)

Molecular formula (neutral free acid): C16H19N2O15P3

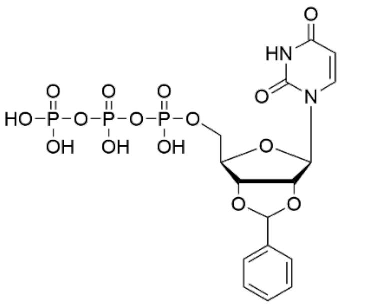

S028 2′,3′-O-(2-methoxycarbonyl)ethylidene-UTP (6d)

Molecular formula (neutral free acid): C13H19N2O17P3

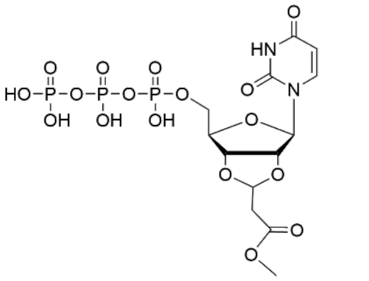

S029 2′,3′-O-isopropylidene-CTP (12a)

Molecular formula (neutral free acid): C12H20N3O14P3

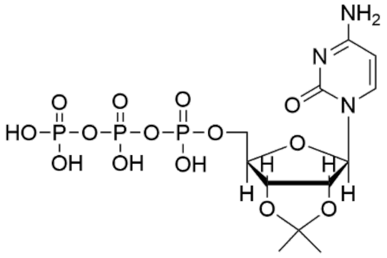

S030 2′,3′-O-isopropylidene-GTP (12b)

Molecular formula (neutral free acid): C13H20N5O14P3

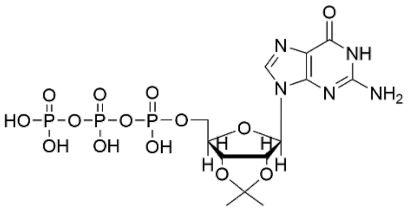

S031 2′,3′-O-cyclohexylidene-ATP (13)

Molecular formula (neutral free acid): C16H24N5O13P3

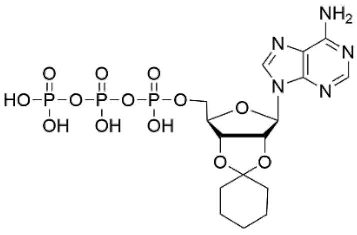

S032 3′-triGalNAc-LNA-TTP (4)

Molecular formula (neutral free acid): C77H128N13O47P3

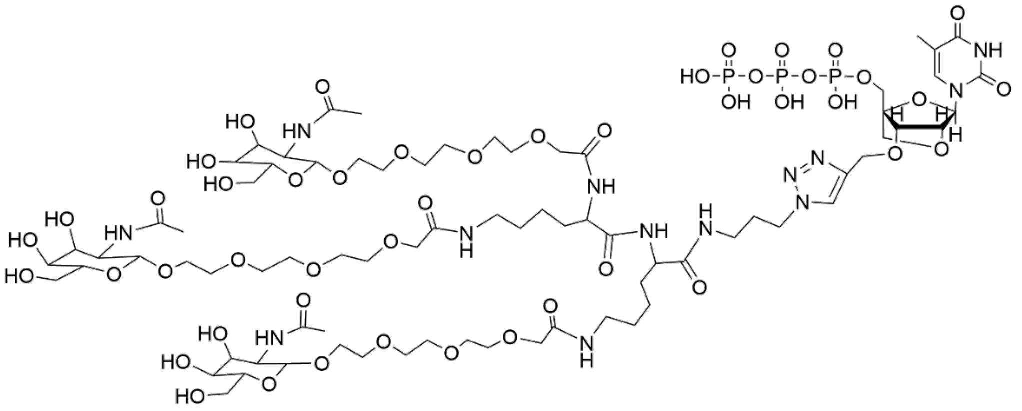

S033 GUTP (glycine-modified UTP)

Molecular formula (neutral free acid): C12H21N4O16P3

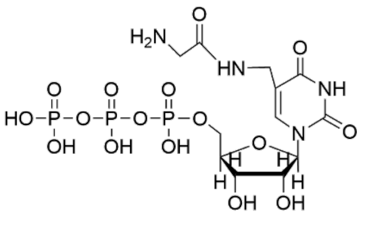

S034 2′,3′-O-isopropylidene-ATP (12c)

Molecular formula (neutral free acid): C13H20N5O13P3

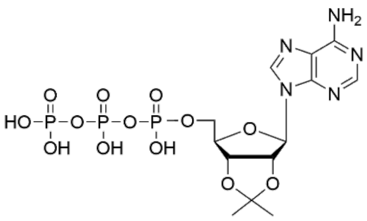

S035 2′,3′-O-isopropylidene-ITP (12d)

Molecular formula (neutral free acid): C13H19N4O14P3

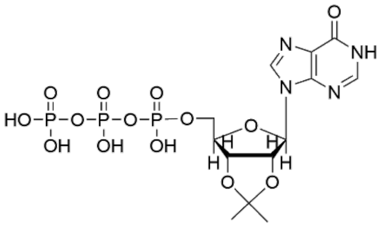

S036 2′,3′-bis-O-acetyl-UTP (4a)

Molecular formula (neutral free acid): C13H19N2O17P3

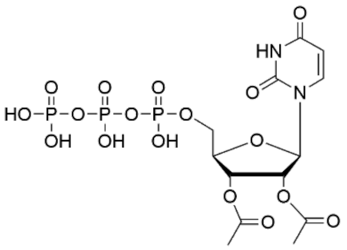

S037 2′,3′-bis-O-benzoyl-UTP (4b)

Molecular formula (neutral free acid): C23H23N2O17P3

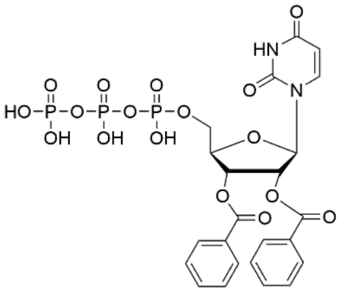

S038 2′,3′-O-trinitrophenyl-UTP (TNP-UTP; 6e)

Molecular formula (neutral free acid): C15H16N5O21P3

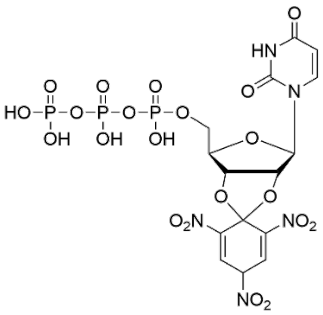

S039 2′-fluoro-2′-deoxy-ATP

Molecular formula (neutral free acid): C10H15FN5O12P3

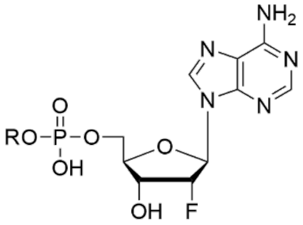

S040 2′-fluoro-2′-deoxy-UTP

Molecular formula (neutral free acid): C9H14FN2O14P3

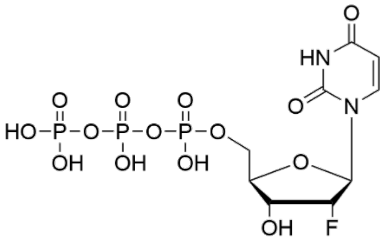

S041 2′-fluoro-2′-deoxy-GTP

Molecular formula (neutral free acid): C10H15FN5O13P3

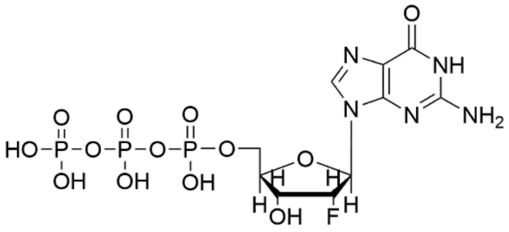

S042 2′-fluoro-2′-deoxy-CTP

Molecular formula (neutral free acid): C9H15FN3O13P3

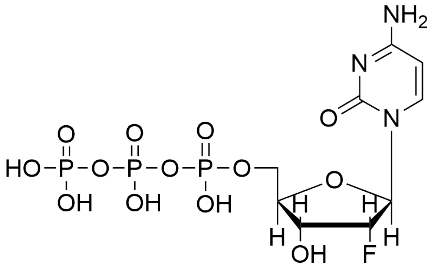

S043 2′-O-methyl-ATP

Molecular formula (neutral free acid): C11H18N5O13P3

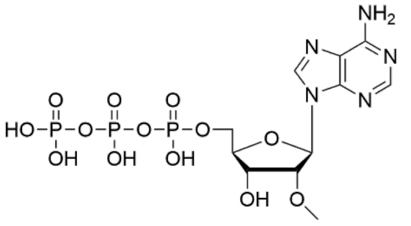

S044 2′-O-methyl-GTP

Molecular formula (neutral free acid): C11H18N5O14P3

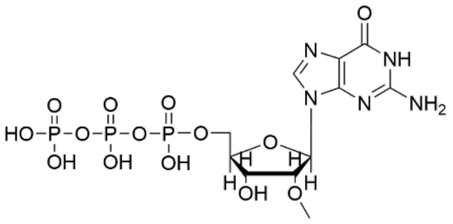

S045 2′-O-methyl-CTP

Molecular formula (neutral free acid): C10H18N3O14P3

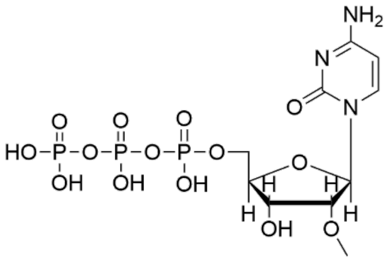

S046 α-PS-ATP

Molecular formula (neutral free acid): C10H16N5O12P3S

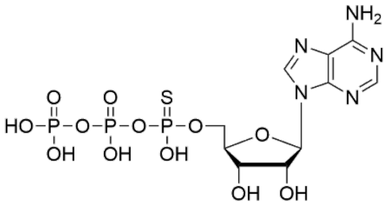

S047 α-PS-UTP

Molecular formula (neutral free acid): C9H15N2O14P3S

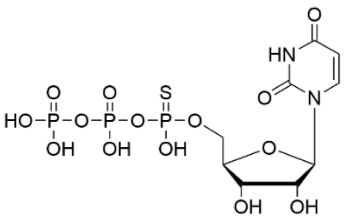

S048 α-PS-GTP

Molecular formula (neutral free acid): C10H16N5O13P3S

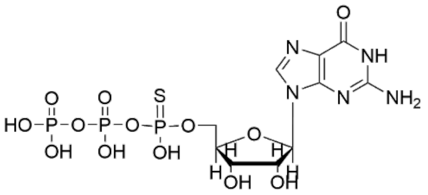

S049 α-PS-CTP

Molecular formula (neutral free acid): C9H16N3O13P3S

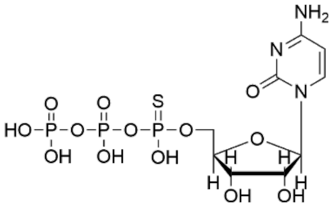

S050 N^6-propargyl-ATP

Molecular formula (neutral free acid): C13H18N5O13P3

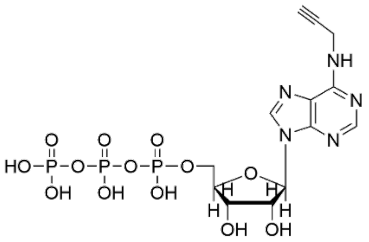

S051 2-ethynyl-ATP

Molecular formula (neutral free acid): C12H16N5O13P3

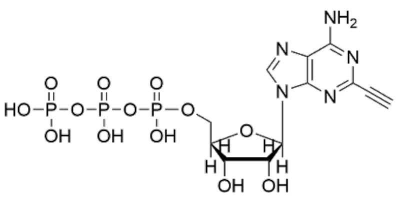

S052 dATP

Molecular formula (neutral free acid): C10H16N5O12P3

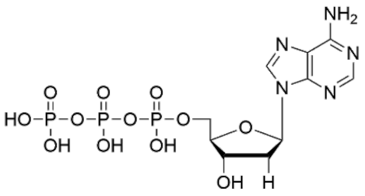

S053 dTTP

Molecular formula (neutral free acid): C10H17N2O14P3

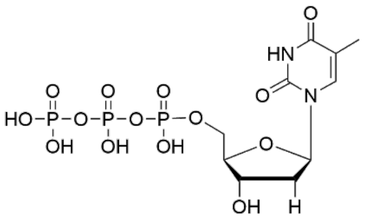

S054 dGTP

Molecular formula (neutral free acid): C10H16N5O13P3

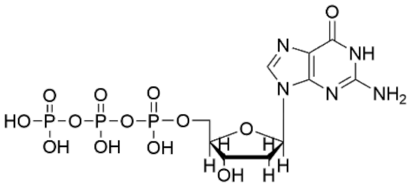

S055 dCTP

Molecular formula (neutral free acid): C9H16N3O13P3

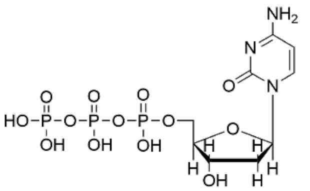

S056 3′-O-allyl-ATP

Molecular formula (neutral free acid): C13H20N5O13P3

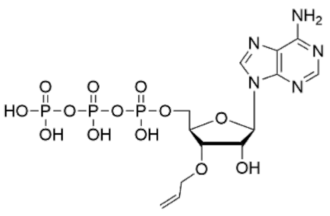

S057 3′-O-allyl-UTP

Molecular formula (neutral free acid): C12H19N2O15P3

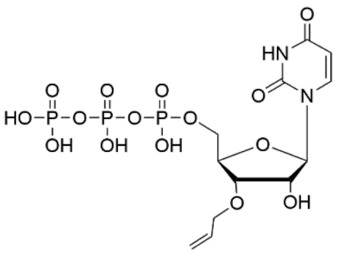

S058 3′-O-allyl-GTP

Molecular formula (neutral free acid): C13H20N5O14P3

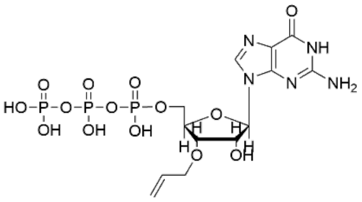

S059 3′-O-allyl-CTP

Molecular formula (neutral free acid): C12H20N3O14P3

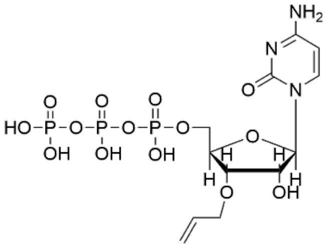

S060 3′-O-allyl-2′-fluoro-2′-deoxy-ATP

Molecular formula (neutral free acid): C13H19FN5O12P3

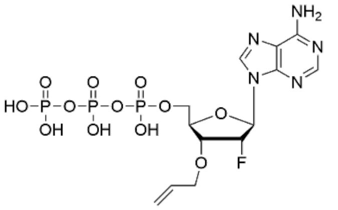

S061 3′-O-allyl-2′-fluoro-2′-deoxy-UTP

Molecular formula (neutral free acid): C12H18FN2O14P3

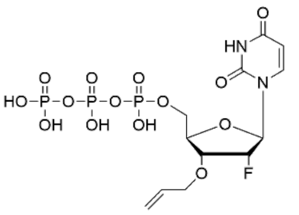

S062 3′-O-allyl-2′-fluoro-2′-deoxy-GTP

Molecular formula (neutral free acid): C13H19FN5O13P3

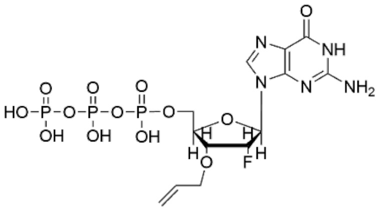

S063 3′-O-allyl-2′-fluoro-2′-deoxy-CTP

Molecular formula (neutral free acid): C12H19FN3O13P3

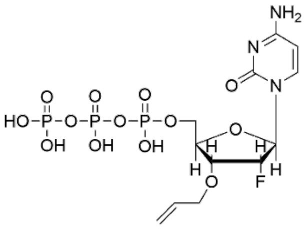

S064 3′-O-allyl-2′-O-methyl-ATP

Molecular formula (neutral free acid): C14H22N5O13P3

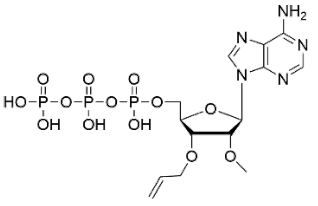

S065 3′-O-allyl-2′-O-methyl-UTP

Molecular formula (neutral free acid): C13H21N2O15P3

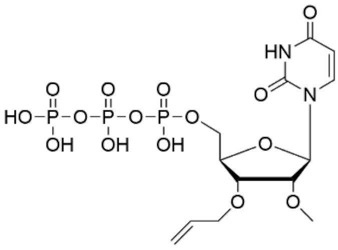

S066 3′-O-allyl-2′-O-methyl-GTP

Molecular formula (neutral free acid): C14H22N5O14P3

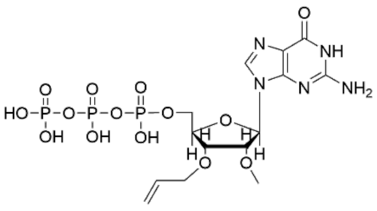

S067 3′-O-allyl-2′-O-methyl-CTP

Molecular formula (neutral free acid): C13H22N3O14P3

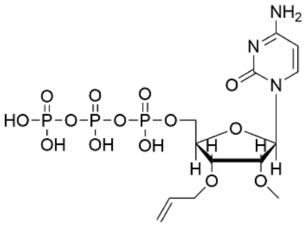

S068 3′-O-allyl-ATP-α-PS

Molecular formula (neutral free acid): C13H20N5O12P3S

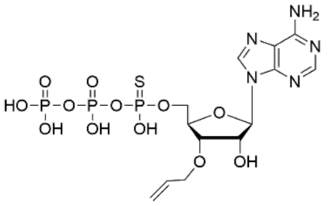

S069 3′-O-allyl-UTP-α-PS

Molecular formula (neutral free acid): C12H19N2O14P3S

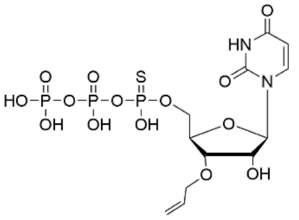

S070 3′-O-allyl-GTP-α-PS

Molecular formula (neutral free acid): C13H20N5O13P3S

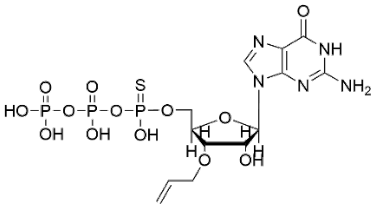

S071 3′-O-allyl-CTP-α-PS

Molecular formula (neutral free acid): C12H20N3O13P3S

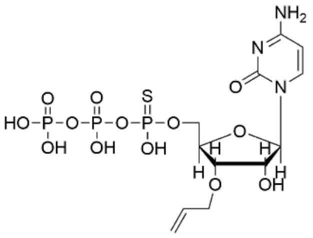

S072 3′-O-propargyl-ATP

Molecular formula (neutral free acid): C13H18N5O13P3

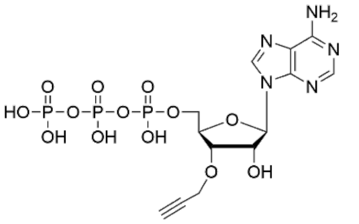

S073 3′-O-propargyl-UTP

Molecular formula (neutral free acid): C12H17N2O15P3

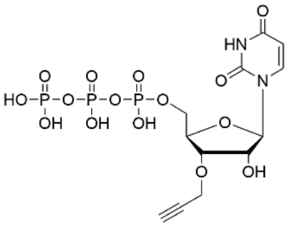

S074 3′-O-propargyl-GTP

Molecular formula (neutral free acid): C13H18N5O14P3

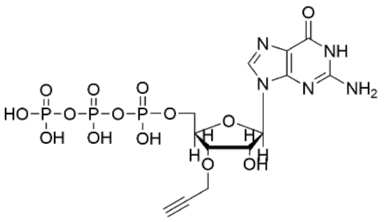

S075 3′-O-propargyl-CTP

Molecular formula (neutral free acid): C12H18N3O14P3

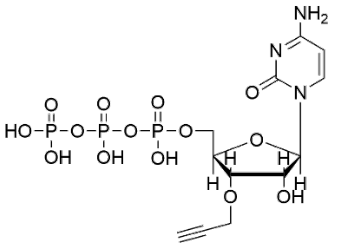

S076 3′-O-azidomethyl-dATP

Molecular formula (neutral free acid): C11H17N8O12P3

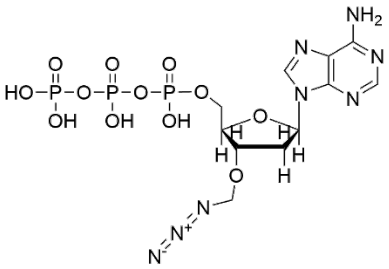

S077 3′-O-azidomethyl-dGTP

Molecular formula (neutral free acid): C11H17N8O13P3

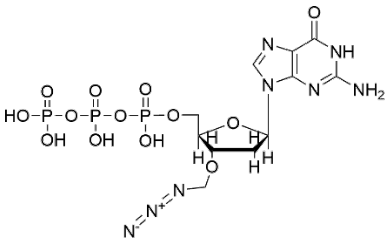

S078 3′-O-azidomethyl-dCTP

Molecular formula (neutral free acid): C10H17N6O13P3

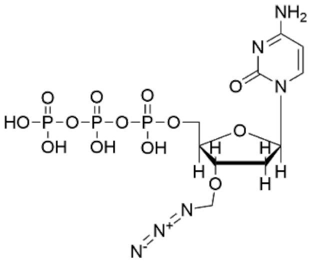

S079 3′-O-azidomethyl-ATP-α-PS

Molecular formula (neutral free acid): C11H17N8O12P3S

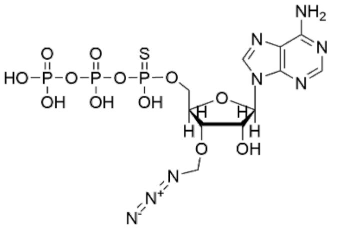

S080 3′-O-azidomethyl-UTP-α-PS

Molecular formula (neutral free acid): C10H16N5O14P3S

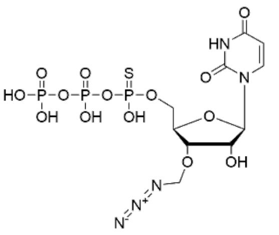

S081 3′-O-azidomethyl-CTP-α-PS

Molecular formula (neutral free acid): C10H17N6O13P3S

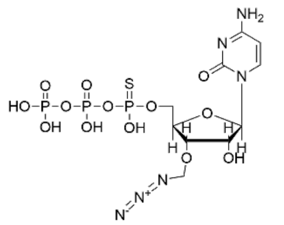

S082 3′-O-allyl-ITP

Molecular formula (neutral free acid): C13H19N4O14P3

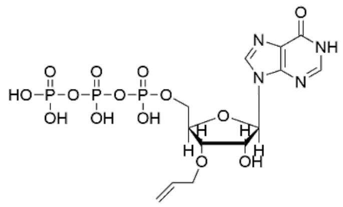

Supplement: Supplementary file 1 [file biomolecules-16-01535-s001.zip › Figure S1.pdf]
